# Genome-Wide Assessment of AU-Rich Elements by the ARE*Score* Algorithm

**DOI:** 10.1371/journal.pgen.1002433

**Published:** 2012-01-05

**Authors:** Milan Spasic, Caroline C. Friedel, Johanna Schott, Jochen Kreth, Kathrin Leppek, Sarah Hofmann, Sevim Ozgur, Georg Stoecklin

**Affiliations:** 1 Helmholtz Junior Research Group Posttranscriptional Control of Gene Expression, German Cancer Research Center, DKFZ-ZMBH Alliance, Heidelberg, Germany; 2 Institute for Informatics, Ludwig-Maximilians-Universität (LMU) München, Munich, Germany; Stanford University School of Medicine, United States of America

## Abstract

In mammalian cells, AU-rich elements (AREs) are well known regulatory sequences located in the 3′ untranslated region (UTR) of many short-lived mRNAs. AREs cause mRNAs to be degraded rapidly and thereby suppress gene expression at the posttranscriptional level. Based on the number of AUUUA pentamers, their proximity, and surrounding AU-rich regions, we generated an algorithm termed ARE*Score* that identifies AREs and provides a numerical assessment of their strength. By analyzing the ARE*Score* distribution in the transcriptomes of 14 metazoan species, we provide evidence that AREs were selected for in several vertebrates and *Drosophila melanogaster*. We then measured mRNA expression levels genome-wide to address the importance of AREs in SL2 cells derived from *D. melanogaster* hemocytes. Tis11, a zinc finger RNA–binding protein homologous to mammalian tristetraprolin, was found to target ARE–containing reporter mRNAs for rapid degradation in SL2 cells. *Drosophila* mRNAs whose expression is elevated upon knock down of Tis11 were found to have higher ARE*Scores*. Moreover high ARE*Scores* correlate with reduced mRNA expression levels on a genome-wide scale. The precise measurement of degradation rates for 26 *Drosophila* mRNAs revealed that the ARE*Score* is a very good predictor of short-lived mRNAs. Taken together, this study introduces ARE*Score* as a simple tool to identify ARE–containing mRNAs and provides compelling evidence that AREs are widespread regulatory elements in *Drosophila*.

## Author Summary

Many genes are regulated at the posttranscriptional level by factors that influence the stability of the messenger RNA. In mammals, AU-rich elements are known to cause rapid degradation of messenger RNAs and thereby suppress gene expression. In order to identify such elements on a genome-wide scale, we developed a bioinformatic tool with which we can score messenger RNAs for the presence of AU-rich elements. Using the ARE*Score* algorithm, we observe that AU-rich elements correlate with reduced messenger RNA stability and expression levels. We then used the ARE*Score* to compare the transcriptomes of 14 metazoan species and found that messenger RNAs with high ARE*Scores* are enriched in several vertebrates and the fruit fly *Drosophila melanogaster*. We identified messenger RNAs whose levels are regulated by the *Drosophila* Tis11 protein, which binds to AU-rich elements. Our study introduces the ARE*Score* as a means to globally assess AU-rich elements and predict short-lived messenger RNAs. Furthermore, it demonstrates the regulatory role of AU-rich elements in suppressing gene expression by accelerating messenger RNA degradation in *D. melanogaster* cells.

## Introduction

Gene expression is extensively regulated at both transcriptional and posttranscriptional levels. In the cytoplasm, numerous mechanisms act on mRNAs to ensure their proper localization, translation and stability [1]. Together with the rate of transcription, the lifespan of an mRNA is a key determinant of the level at which any given gene is expressed. Half-lives differ widely between transcripts, ranging in human cells from 5 minutes to >10 hours [2], [3]. In yeast, mRNAs are degraded more rapidly and their half-lives range from 3 to >90 minutes [4].

AU-rich elements (AREs) are well-characterized *cis*-acting regulatory sequences that strongly accelerate the degradation of mammalian mRNAs. AREs were initially discovered in 3′ untranslated regions (UTRs) of short-lived transcripts encoding cytokines [5], [6], and since have been proposed to reside in 5–8% of all transcripts [7]. However, the frequency of functional AREs in a given cell type is certainly lower because genome-wide measurements of mRNA decay rates showed that the presence of AU-rich sequences correlates only to a limited extent with rapid mRNA decay: In primary human T-cells, only about 25% of mRNAs with AU-rich sequences were found to decay rapidly [2], and in the hepatocellular carcinoma cell line HepG2 this proportion was only 10–15% [8].

Although there is no strict consensus sequence for AREs, the following key motifs have been identified: AUUUA pentamers that frequently occur in multiple copies, which may overlap or localize in close proximity [6], [9]; a related nonameric motif UUAUUUAUU or UUAUUUA(U/A)(U/A), which is strongly linked to rapid mRNA decay [10], [11], [12], [13]; and a generally U-rich or AU-rich context required for maximum efficiency of either pentamers or nonamers [9]. AREs can be distinguished according to different deadenylation kinetics, which gave rise to a widely used classification published by Shyu *et al.* [14]. Class I AREs (e.g. c-*myc*, c-*fos*) contain a few scattered pentamers within a larger U- or AU-rich context, and mediate synchronous deadenylation indicative of a distributive exoribonuclease. Class II AREs (e.g. GM-CSF, IL-3 and TNFα) have a cluster of 4–7 partially overlapping pentamers within a U-rich context, and mediate asynchronous deadenylation indicative of a processive exoribonuclease. Class III AREs (e.g., c-*jun*) lack pentamers and have been less well characterized. Khabar *et al.* proposed an alternative classification of AREs into five groups based on the number of overlapping AUUUA pantamers [15]. This classification has been used to mine databases for the occurrence of ARE-regulated genes [7], yet the functional implication of this classification has not been thoroughly tested.

ARE-mediated mRNA decay (AMD) depends on specific RNA-binding proteins (BPs) that recognize AREs and target the mRNA for rapid degradation [16]. The Tis11 zinc finger proteins are ARE-BPs with a major role in AMD. The mammalian Tis11 family comprises TTP [17], BRF1 [18] and BRF2 [19], all of which are potent inducers of mRNA degradation. These proteins share a highly conserved tandem C_3_H zinc finger domain required for RNA binding. TTP is the best characterized member of this family and acts as a suppressor of inflammation in mice by controlling the expression of tumor necrosis factor-α (TNFα) [17]. Further studies showed that TTP causes the degradation of many additional mRNAs related to the immune response (reviewed in [20]). TTP induces the degradation of its target mRNAs by recruiting the components of the general RNA degradation machinery such as the exosome [21], the decapping complex [22] and the Ccr4-Caf1-Not deadenylation complex [23]. Moreover, TTP is regulated through phosphorylation by the p38-MAPK – MK2 kinase cascade. Direct phosphorylation by MK2 causes binding of 14-3-3 adaptor proteins and decreases the activity of TTP [24], [25] by interfering with the ability of TTP to recruit the Ccr4-Caf1-Not deadenylation complex [26], [27]. In turn, the protein phosphatase 2A dephosphorylates TTP and thereby activates AMD [28].

Very little is known about AMD in *Drosophila melanogaster*. So far, only the mRNAs encoding CecA1 and bnl were shown to contain a functional ARE [29], [30], [31]. The CecA1 ARE binds to Tis11, the homologue of TTP in *D. melanogaster*, which in turn promotes rapid degradation of CecA1 mRNA by enhancing deadenylation [29]. Interestingly, expression of mammalian TTP could compensate for the knock down of Tis11 in *Drosophila* cells [30], suggesting evolutionary conservation. While the regulation of CecA1 mRNA degradation has been well characterized, there is no experimental study addressing more generally the role of AMD in *D. melanogaster*.

Here we report the development of ARE*Score*, a software application by which mRNAs can be assessed for the presence of AREs. After validating the ARE*Score* using half-life measurements of human and mouse mRNAs, the transcriptome-wide ARE*Score* distribution was analyzed across 14 metazoan species. The ARE*Score* was then applied to the analysis of AMD in *Drosophila* SL2 cells. By combining biochemical and bioinformatic approaches, we provide evidence for a specific set of mRNAs regulated by Tis11, and for the broader role of AREs in controlling mRNA degradation in *D. melanogaster*.

## Results

### ARE*Score* provides a numerical assessment for AREs

**Figure 1:**
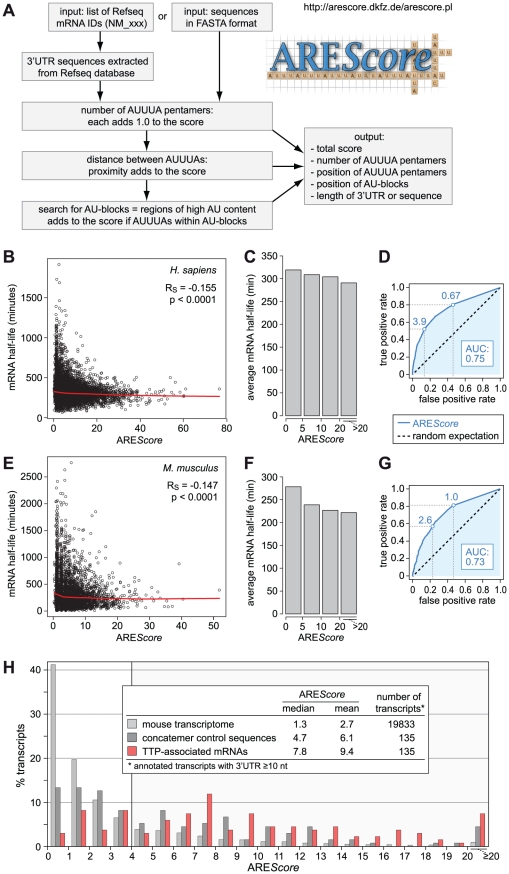
Numerical assessment of AREs using the ARE*Score* algorithm. (A) The ARE*Score* is based on counting the number of AUUUA pentamers per 3′UTR or sequence. The proximity between pentamers and the occurrence of pentamers within larger AU-blocks adds to the score. Sequences to be analyzed can be entered either as a list of Refseq IDs or in FASTA format. A web-based version of the ARE*Score* algorithm is available under http://arescore.dkfz.de/arescore.pl. (B) Relationship between ARE*Score* and genome-wide mRNA half-lives from a study in human DG75 B-cells [32]. The red curve corresponds to non-linear lowess regression, R_S_ to the Spearman rank correlation coefficient. (C) mRNAs depicted in panel B were grouped according to their ARE*Score* and set in relation to the average half-life in each group. (D) ROC analysis was applied to the data in panel B, testing every possible ARE*Score* value for its ability to discriminate the 10% most short-lived mRNAs from the 10% most long-lived mRNAs. AUC, area under the curve. Maximum ARE*Scores* with a true positive rate of at least 0.5 and 0.8, respectively, are indicated by dotted lines. (E) Relationship between ARE*Score* and genome-wide mRNA half-lives from a study in mouse NIH3T3 fibroblasts [33]. Analysis was performed as in panel E. (F) mRNAs depicted in panel E were grouped according to their ARE*Score*, and set in relation to the average half-life in each group. (G) ROC analysis for the data in panel E, as in panel D. (H) ARE*Score* distribution of TTP-associated mRNAs in mouse RAW264.7 macrophages from an RNA-IP study [13]. ARE*Score* frequencies are plotted for 135 mRNAs that were enriched by immunoprecipitation of TTP. As controls, ARE*Score* frequencies are shown for a group of 135 randomly concatenated 3′UTR sequences matching in size to the TTP-associated 3′UTRs, and for the 3′UTRs ≥10 nt in size of all annotated mouse mRNAs.

With the aim to identify genes containing AREs in any given set of sequences, we developed an algorithm termed ARE*Score*, schematically depicted in Figure 1A. Its purpose is to provide a numerical measure of the potential strength of an ARE, and assess the occurrence of AREs on a transcriptome-wide level. The ARE*Score* is based on quantifying three typical features of AREs: the number of AUUUA pentamers, the proximity between pentamers, and the presence of a region with high AU content surrounding AUUUA pentamers. The UUAUUUA(U/A)(U/A) nonamer was not counted as a separate parameter because it largely corresponds to two overlapping AUUUA pentamers or a pentamer within a region of high AU content. The algorithm first counts AUUUA pentamers and attributes a fixed value of 1 for each pentamer to generate a basal score. It then calculates the distance between neighboring pentamers, and adds a value to the basal score if pentamers are close to each other. Likewise, a value is added if pentamers are located within a region of high AU content, herein termed an AU-block. To increase the flexibility of ARE*Score*, users can change the values that are added to the basal score, and alter the settings that define an AU-block. Thereby users can adapt the algorithm to their needs and particular questions. A web-based version of ARE*Score* is available at http://arescore.dkfz.de/arescore.pl.

To validate the algorithm, we calculated the ARE*Score* for every human mRNA in the RefSeq database with a 3′UTR length ≥10 nucleotides (nt), whereby many falsely annotated 3′UTRs could be excluded from the analysis. In Figure 1B, the ARE*Score* was then compared to previously measured mRNA half-lives in human DG75 B-cells [32]. The ARE*Score* shows a slight, but statistically highly significant, negative correlation with mRNA half-life (Spearman rank correlation coefficient R_S_ = −0.155, p<0.0001). The correlation was more apparent when mRNAs were classified into groups with similar ARE*Scores* and the average half-life was plotted for each group (Figure 1C). We then used Receiver Operating Characteristic (ROC) analysis to assess the predictive power of the ARE*Score* in this dataset (Figure 1D). Every possible ARE*Score* value was tested for its ability to discriminate the 10% most short-lived mRNAs from the 10% most long-lived ones. For instance, mRNAs with an ARE*Score* ≥3.9 make up 53% of the short-lived mRNAs (true positive rate), but only 14% of the long-lived mRNAs (false positive rate). By plotting true positive rate against false positive rate for every possible ARE*Score*, the ROC curve is obtained. The area under this curve (AUC) corresponds to the probability that a random short-lived mRNA has a higher ARE*Score* than a random long-lived mRNA. With a value of 0.75, the AUC is well above that of a random predictor (AUC = 0.5).

In a similar manner, we compared the ARE*Score* of mouse mRNAs with half-lives measured previously in mouse NIH3T3 fibroblasts [33]. This analysis again showed a weak but highly significant negative correlation between ARE*Score* and mRNA half-life (Figure 1E, R_S_ = −0.147, p<0.0001, and Figure 1F). With an AUC of 0.73, the ROC curve of the mouse dataset (Figure 1G) is very similar to the curve of the human dataset (Figure 1D). Taken together, the comparison of ARE*Scores* with genome-wide measurements of mRNA half-lives showed that mRNAs with a high ARE*Score* are more likely to be short-lived, both in human and mouse cell lines.

To further validate the ARE*Score*, we analyzed a set of transcripts that we had previously identified as TTP-associated mRNAs in mouse macrophages using RNA-immunoprecipitation [13]. Figure 1H shows that the ARE*Score* is very high among the 135 TTP-associated mRNAs (median 7.8) compared to the entire mouse transcriptome (median 1.3) or a more stringent control set of randomly chosen, concatenated 3′UTR sequences (median 4.65) whose lengths were matched to the lengths of the TTP-associated 3′UTRs. To test whether the ARE*Score* distribution of the TTP-associated mRNAs was statistically different from the controls, we compared the frequency of mRNAs with an ARE*Score* <4 and ≥4 in 2×2 contingency tables (Tables S1 and S2). P-values were calculated either by χ^2^-test or Fisher's exact test, and found to be <0.0001 for both comparisons. Thus, the ARE*Scores* of the TTP-associated mRNAs were significantly higher than the ARE*Scores* of both control groups. This confirmed that the ARE*Score* is a useful tool to identify ARE-containing mRNAs.

### AREs are more abundant in vertebrate and arthropod genomes

**Figure 2:**
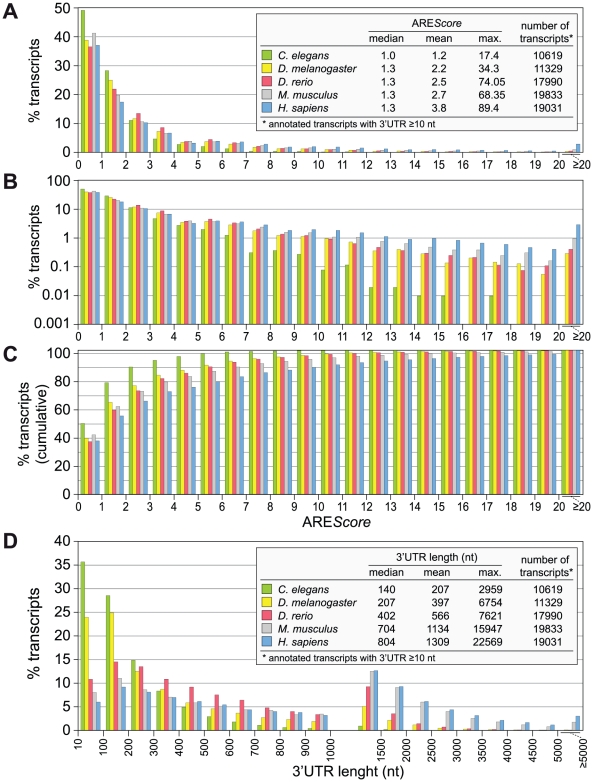
ARE*Score* distribution in different metazoa. (A) The ARE*Score* was determined for every annotated transcript with a 3′UTR length ≥10 nt, for *Caenorhabditis elegans*, *Drosophila melanogaster*, *Danio rerio*, *Mus musculus* and *Homo sapiens*. mRNAs were grouped according to ARE*Scores* of 0–0.99, 1–1.99, 2–2.99, etc. The graph shows the frequency of mRNAs in each group using a linear scale. (B) The same distribution as in panel A is shown on a logarithmic scale to better visualize the low abundant mRNAs with high ARE*Scores*; frequencies of 0 were omitted from the graph. (C) The same distribution as in panel A is depicted using cumulative frequencies on a linear scale. (D) The 3′UTR length distribution was plotted for the same set of transcripts. mRNAs were grouped according to 3′UTR lengths of 10–99, 100–199, 200–299, … 1000–1499, 1500–1999, … 4500–4999, and ≥5000 nt.

Having the ARE*Score* at hand as a numerical tool to estimate the abundance and strength of AREs in any given genome, we calculated the ARE*Score* of all annotated transcripts with a 3′UTR length ≥10 nt for *Homo sapiens* and four important metazoan model organisms, *Caenorhabditis elegans*, *D. melanogaster*, *Danio rerio*, and *Mus musculus*. The analysis shows that in all five species, the vast majority of mRNAs have a score below 4 (Figure 2A). Differences became apparent when frequencies were plotted on a logarithmic scale (Figure 2B). The highest ARE*Score* is 17.4 in *C. elegans* and 34.3 in *D. melanogaster*, whereas in the two mammalian species ARE*Scores* go beyond 60. These differences are also visible in the plot of cumulative frequencies (Figure 2C), which shows the highest prevalence of low ARE*Scores* in *C. elegans* and of high ARE*Scores* in *H. sapiens*. It was interesting to note that the 3′UTR length follows a similar pattern (Figure 2D), with *C. elegans* having the by far shortest 3′UTRs (median: 140 nt), followed by *D. melanogaster* (207 nt) and *D. rerio* (402 nt), and considerably longer 3′UTRs in the two mammalian species (704 nt in mouse, 804 in man). This analysis shows that mRNAs with high ARE*Scores* as well as long 3′UTRs are more abundant in the two mammalian species, which likely reflects the need for additional elements regulating gene expression.

### Evolutionary selection for AREs

**Figure 3:**
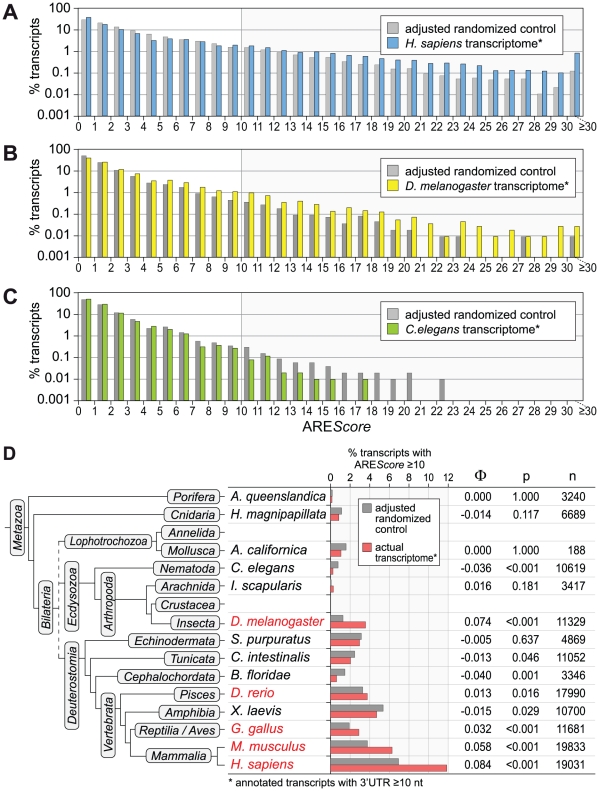
ARE*Score* distribution in comparison to randomized controls. (A) The ARE*Score* distribution of the *H. sapiens* transcriptome (every annotated transcript with a 3′UTR length ≥10 nt) was compared to a fully adjusted, randomized control set of sequences with identical lengths and A/T/G/C-content. Percentage of transcripts is depicted on a logarithmic scale. (B) The same analysis was done with the *D. melanogaster* transcriptome, as in panel A. Frequencies of 0 were omitted from the graph. (C) The same analysis was done with the *C. elegans* transcriptome, as in panel B. (D) The frequency of mRNAs with an ARE*Score* ≥10 in the actual transcriptome of 14 species was compared to the frequency in fully adjusted, randomized control sequences. The analysis was carried out for *Amphimedon queenslandica* (demosponge), *Hydra magnipapillata* (freshwater polyp), *Aplysia californica* (California sea hare), *Caenorhabditis elegans* (roundworm), *Ixodes scapularis* (deer tick), *Drosophila melanogaster* (fruit fly), *Strongylocentrotus purpuratus* (purple sea urchin), *Ciona intestinalis* (vase tunicate), *Branchiostoma floridae* (Florida lancelet), *Danio rerio* (zebrafish), *Xenopus laevis* (African clawed frog), *Gallus gallus* (chicken), *Mus musculus* (common house mouse) and *Homo sapiens* (man). The Φ coefficient serves as a measure for how strongly ARE*Scores* ≥10 are associated with the actual transcriptome as compared to the randomized control. P-values were calculated by χ^2^-test, n represents the number of transcripts. Species labeled in red show a significant enrichment of mRNAs with ARE*Scores* ≥10.

To test whether AREs are truly enriched in any of the transcriptomes we analyzed, we compared the ARE*Score* distribution in different species with sets of randomized sequences that have identical A/T/G/C contents and length distributions (Figure 3 and Figure S1). This comparison revealed that mRNAs with high ARE*Scores* (≥10) are overrepresented in the transcriptome of *H. sapiens* (Figure 3A). In *D. melanogaster*, the enrichment already starts at an ARE*Score* of 4 (Figure 3B), whereas there is no enrichment of mRNAs with higher ARE*Scores* in *C. elegans* (Figure 3C). We then expanded this analysis to the transcriptomes of 11 additional species, covering most of the major branches of metazoan evolution (Figure S1). Only for Annelida and Crustacea, no properly annotated transcriptomes were available. In the 14 species analyzed, the frequency of mRNAs with an ARE*Score* ≥10 was compared to the frequency of ARE*Scores* ≥10 in sets of randomized control sequences (Figure 3D). mRNAs with an ARE*Score* ≥10 were found to be overrepresented in the transcriptomes of *H. sapiens*, *M musculus*, *Gallus gallus* (chicken), *Danio rerio* (zebrafish) and *D. melanogaster*. This is reflected by a positive Φ coefficient, a measure for how strongly ARE*Scores* ≥10 are associated with the actual transcriptome as compared to the randomized control. In all these cases, the difference was significant as determined by χ^2^-test. mRNAs with an ARE*Score* ≥10 were also more abundant in *Ixodes scapularis* (deer tick), although in this case the difference was statistically not significant. In the 8 other species analyzed, mRNAs with an ARE*Score* ≥10 were less abundant than in the randomized control sequences. Thus, our analysis suggested that AREs were selected for during the evolution of several vertebrate species (*Xenopus laevis* being the exception) as well as *D. melanogaster*.

### ARE*Score* of Tis11-sensitive mRNAs in *Drosophila* cells

**Figure 4:**
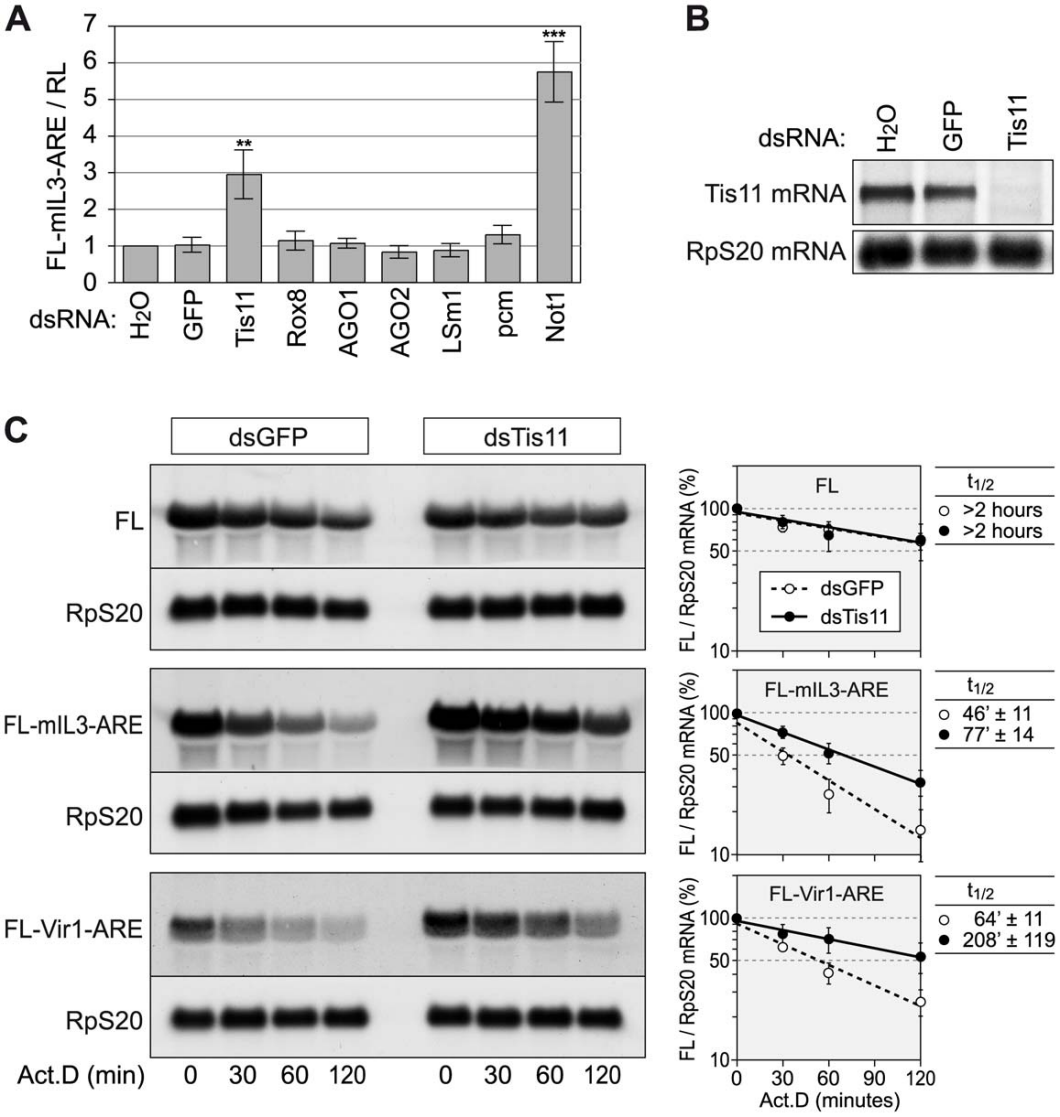
Tis11 mediates rapid mRNA degradation in *D. melanogaster* SL2 cells. (A) To knock down the expression of several mRNA decay factors, SL2 cells were treated with dsRNA (12.5 µg/ml) over a period of 4 days. For control, cells were treated with water alone, or with a dsRNA targeting GFP. On day 3, cells were transfected with a firefly luciferase (FL) reporter containing the ARE of mouse IL3 (pRp128-FL-mIL3-ARE) together with pRp128-RL encoding *Renilla* luciferase (RL). FL and RL activities were measured on day 5 and normalized to the water control. The graph shows average FL-mIL3-ARE/RL ratios ± SD based on 4–9 biological repeats. In comparison to the GFP control, dsRNAs targeting Tis11 or Not1 cause a highly significant increase with p-values of (**) 1.6×10^−6^ and (***) 3.3×10^−9^, respectively. (B) The Tis11 knock down efficiency was examined after treatment of SL2 cells with dsRNA for 5 days. Total RNA was extracted, and 8 µg per sample were subjected to Northern blot analysis using a probe against Tis11 mRNA. Ribosomal protein RpS20 mRNA serves as a loading control. (C) To measure reporter mRNA stability, SL2 cells were treated with dsRNAs against GFP or Tis11 over a period of 4 days, and transiently transfected with Ac5-FL, Ac5-FL-mIL3-ARE or Ac5-FL-Vir1-ARE on day 3. On day 5, cells were treated with actinomycin D (5 µg/ml), and total RNA was extracted after 0, 30, 60 and 120 minutes. Per sample, 4–5 µg of RNA were subjected to Northern blot analysis. mRNA signal intensities from three (FL) or four (IL3, Vir1) biological repeat experiments were quantified, and the average FL/RpS20 ratio ± SE was plotted against time in the panels on the right side. mRNA half-lives are given as average values ± SE.

Given that we found AREs to be overrepresented in the *D. melanogaster* transcriptome (Figure 3B) and that little is known about the general importance of AMD in this organism, we decided to experimentally address the functional relevance of the ARE*Score* in *Drosophila*. We first established an assay to measure AMD in *D. melanogaster* SL2 cells by generating firefly luciferase (FL) reporter genes containing the ARE of mouse interleukin (IL)-3 in the 3′UTR (Figure S2A, IL3 ARE sequence depicted in Figure S3). Expression of the FL reporter gene was found to be strongly suppressed by the IL3 ARE in SL2 cells, both at the protein (luciferase activity) and mRNA level, and suppression was due to accelerated degradation of the reporter mRNA (Figure S2B–S2D). We then tested several factors for their involvement in *Drosophila* AMD by knocking down their expression using dsRNAs. Whereas knock down (kd) of Tis11 and Not1, a core protein of the cytoplasmic Ccr4-Caf1-Not deadenylation complex, caused elevated expression of the ARE-reporter, other proteins such as Rox8, AGO1, AGO2, LSm1 and pcm did not affect reporter gene expression (Figure 4A).

**Figure 5:**
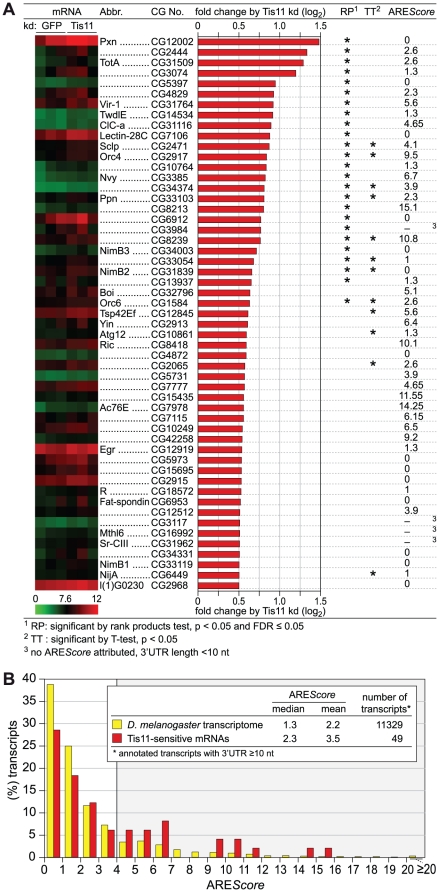
Analysis of Tis11-sensitive mRNAs in *D. melanogaster* SL2 cells. (A) *D. melanogaster* SL2 cells were treated over a period of 4 days with 12.5 µg/ml dsRNA in order to knock down Tis11, or with dsRNA targeting GFP as a control. Total RNA was extracted from three biological repeats for microarray analysis using the Affymetrix Drosophila Genome 2.0 array. After normalization of the signal intensities using Robust Multi-array Analysis (RMA), the fold change of expression by Tis11 kd (signal in Tis11 kd/signal in GFP kd) was calculated. The list shows all 53 mRNAs with a log2-transformed fold change of >0.5, i.e. a fold change of >1.41. Statistical significance was determined by rank products (RP) test and independently by Student's T-test (TT). A heat map of the signal intensities in the three biological repeats is provided on the left side, the ARE*Score* is shown on the right side. (B) The ARE*Score* distribution is depicted for 49 out of the 53 Tis11-sensitive mRNAs identified in panel A. Only mRNAs with an annotated 3′UTR length ≥10 nt were included. The ARE*Score* distribution of the entire *D. melanogaster* transcriptome serves as the control group.

Since *Drosphila* Not1 is important for mRNA deadenylation in general [34], [35], we focused on Tis11 as an ARE-specific RNA binding protein. Our goal was to examine the ARE*Score* of mRNAs regulated by Tis11. We first confirmed that the dsRNA against Tis11 potently suppressed the expression of Tis11 mRNA (Figure 4B), and that Tis11 kd stabilizes the FL-mIL3-ARE reporter mRNA (Figure 4C). To identify *Drosophila* mRNAs regulated by Tis11, we determined the mRNA expression profile in SL2 cells after knocking down Tis11 or, as a control, GFP. Since direct targets of Tis11 are expected to show higher expression levels after Tis11 kd, we concentrated on the 53 mRNAs that we found to be upregulated by a factor of at least 1.41 (0.5 log_2_-transformed) after Tis11 kd in the microarray analysis (Figure 5A). 20 out of these mRNAs were chosen for confirmation by qPCR, and for 18 of them we could verify that Tis11 kd causes a an increase in expression of minimally 1.41-fold (Figure S4), indicating that our microarray dataset was reliable. The Vir1 mRNA, which was strongly upregulated by Tis11 kd (Figure S4), has an ARE*Score* of 5.6 and a readily detectable ARE (Figure S3). Indeed, an FL reporter mRNA containing the ARE of *Drosophila* Vir1 was stabilized by kd of Tis11 (Figure 4C).

Out of the 53 mRNAs sensitive to Tis11 kd, we then determined the ARE*Score* for those 49 transcripts whose annotated 3′UTR length is ≥10 nt. In comparison to the ARE*Score* distribution of the entire *D. melanogaster* transcriptome, the Tis11-sensitive mRNAs showed an increased abundance of ARE*Scores* ≥4 (Figure 5B). By χ^2^-test, this increase was statistically significant with a p-value of 0.0011 (Table S3), suggesting that target mRNAs of *Drosophila* Tis11 share characteristics with mammalian AREs.

### ARE*Score* correlates with *Drosophila* mRNA half-life and expression level

**Figure 6:**
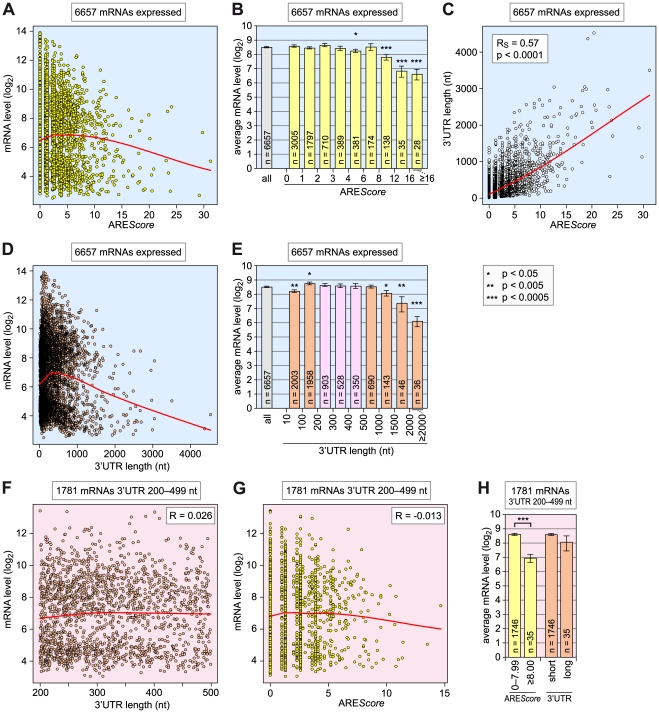
Relationship among ARE*Score*, 3′UTR length, and mRNA expression level. (A) For 6657 *Drosophila* mRNAs with a 3′UTR ≥10 nt, the log_2_-transformed expression levels, as measured by microarray analysis in SL2 cells under control conditions (dsGFP), were plotted against the ARE*Score*. The red curve corresponds to non-linear lowess regression. (B) The 6657 mRNAs were grouped into 9 categories according to their ARE*Score*: 0–0.99; 1–1.99; 2–2.99; 3–3.99; 4–5.99; 6–7.99; 8–11.99; 12–15.99; and ≥16. Average expression levels ± SE were determined for each group and plotted in the graph, the number n of mRNAs within each group is indicated. Asterisks mark statistically significant differences in comparison to the average expression level of all 6657 mRNAs, as determined by Student's T-test. (C) The 3′UTR length of all 6657 mRNAs was plotted against the ARE*Score*. The red curve corresponds to non-linear lowess regression. (D) The log_2_-transformed expression levels of all 6657 mRNAs were plotted against the 3′UTR length. The red curve corresponds to non-linear lowess regression. (E) The 6657 mRNAs were grouped into 9 categories according to their 3′UTR length: 10–99; 100–199; 200–299; 300–399; 400–499; 500–999; 1000–1499; 1500–1999; and ≥2000 nt. Average expression levels ± SE were represented as in panel B. (F) For the 1781 mRNAs with a 3′UTR length between 200 and 499 nt, the log_2_-transformed expression levels were plotted against the 3′UTR length. The red curve corresponds to non-linear lowess regression, R to the Pearson correlation coefficient. (G), For the same 1781 mRNAs, the log_2_-transformed expression levels were plotted against the ARE*Score*. (H) The same 1781 mRNAs were divided into two groups based on ARE*Scores* of 0–7.99 and ≥8. The same mRNAs were also divided into two equally large groups according to the 3′UTR length, and average expression levels ± SE were determined for each group.

After applying the ARE*Score* to the subgroup of Tis11-sensitive mRNAs, we wanted to assess the importance of AREs in regulating *Drosophila* gene expression more generally. If AREs are wide-spread elements that promote mRNA degradation but do not affect transcription, a first prediction is that, on average, mRNAs with a high ARE*Score* should be expressed at lower levels. A second prediction is that these mRNAs should have shorter half-lives. We tested the first prediction by comparing the expression levels of 6657 mRNAs, derived from our microarray analysis in SL2 cells, with their ARE*Scores* (Figure 6A). Indeed, we observed a tendency for mRNAs with high ARE*Scores* to be expressed at lower levels. We further grouped the mRNAs into 9 categories according to their ARE*Score*, and compared the average expression levels of each group to the overall average (Figure 6B). The two groups with very high ARE*Scores* ≥12 showed average expression levels that were more than 3-fold (1.6 log_2_-transformed) below the overall average, and the reduction in expression was already significant above an ARE*Score* of 8.

We then compared the 3′UTR lengths with the ARE*Scores* of all 6657 mRNAs (Figure 6C). As expected, we found a very strong correlation between these two parameters (R_S_ = 0.57, p<0.0001). Thus, it was important to assess whether the 3′UTR length on its own had an influence on mRNA expression levels (Figure 6D and 6E). Two opposing correlations were apparent: mRNAs with very short 3′UTRs <100 nt, and mRNA with long 3′UTRs ≥1000 nt were expressed at significantly reduced levels, whereas mRNAs with 3′UTRs of intermediate length (100–999 nt) showed the highest expression levels. In fact, the 3′UTR length appeared to have a stronger influence on the expression level than the ARE*Score*, as mRNAs with 3′UTRs ≥2000 nt were expressed more than 5-fold (2.4 log_2_-transformed) below the overall average. To examine whether the predictive power of the ARE*Score* is independent of 3′UTR length, we chose to analyze a subgroup of 1781 mRNAs with 3′UTRs between 200 and 499 nt (pink bars in Figure 6E). In this group, the length of the 3′UTR per se does not negatively correlate with mRNA levels (Figure 6F), whereas mRNAs with higher ARE*Scores* do show a trend towards reduced expression levels (Figure 6G). Indeed, the 35 mRNAs that have an ARE*Score* ≥8 within this group had a more than 3-fold (1.6 log_2_-transformed) reduced average expression level compared to the 1746 mRNAs that have an ARE*Score* between 0 and 7.99 (Figure 6H), and this difference was highly significant (p<0.0005). In contrast, the average expression level of the 35 mRNAs with the longest 3′UTRs was only 1.4-fold (0.5 log_2_-transformed) below the expression level of the remaining 1749 mRNAs with the shorter 3′UTRs. From this comparison we concluded that a high ARE*Score* correlates with lower mRNA expression levels independently of the 3′UTR length.

**Figure 7:**
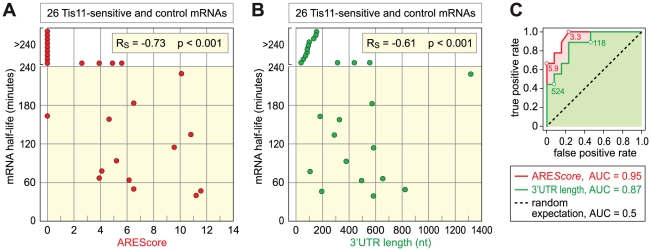
Relationship among ARE*Score*, 3′UTR length, and mRNA half-lives. (A) The half-lives of 26 Tis11-sensitive and control mRNAs, as measured by qPCR in SL2 cells subjected to control GFP dsRNA, were set in relation to their ARE*Score*. Half-lives above 240 minutes could not be determined accurately, and were presented without scale in the white area at the top of the graph. R_S_, Spearman rank correlation coefficient. (B) The half-lives of the same mRNAs were compared to their 3′UTR length. Half-lives above 240 minutes could not be determined accurately, and were presented without scale in the white area at the top of the graph. (C) ROC analysis was applied to the 26 mRNAs, testing the ability of both ARE*Score* and 3′UTR length to discriminate mRNAs with a half-life <140 minutes from mRNAs with a half-life >240 minutes. AUC, area under the curve.

Finally, we tested the second prediction that mRNAs with higher ARE*Scores* should undergo more rapid decay. To this end, we measured the half-lives of 26 mRNAs with high accuracy by qPCR (Table 1, Figure S5). 12 mRNAs were chosen from the group of Tis-11 sensitive mRNAs, and 14 from the large pool of mRNAs that are not affected by Tis11 kd. To cover the entire range, 5 mRNAs had a high ARE*Score* ≥8, 8 mRNAs had a medium ARE*Score* between 4 and 7.99, and 13 had a low ARE*Score* <4. In Figure 7A, we plotted the half-lives of these mRNAs against the ARE*Score*. The most striking observation was that 9 out of 10 mRNAs with an ARE*Score* of 0 degraded very slowly with half-lives >240 minutes. On the other side, the two mRNAs with the highest ARE*Score* (CG115435 from the group of Tis11-sensitive mRNAs and Reck and from the control group) also had the shortest half-lives. In our analysis of 26 mRNAs, the Spearman's rank correlation coefficient R_S_ between the two parameters equals −0.73, and this correlation was highly significant (p<0.001). We also compared the half-lives of these 26 mRNAs with their 3′UTR length (Figure 7B), and found a weaker correlation (R_S_ = −0.61, p<0.001). ROC analysis was then applied to test the ability of both ARE*Score* and 3′UTR length to discriminate labile mRNAs with half-lives <140 minutes from stable mRNAs with half-lives >240 minutes (Figure 7C). The ARE*Score* performed extremely well in this test with an AUC of 0.95, better than 3′UTR length with an AUC of 0.87. Clearly, the ARE*Score* identifies short-lived mRNAs in *D. melanogaster*, showing that AREs are general regulatory elements in this organism.

**Table 1:** *Drosophila* mRNA half-lives in SL2 cells treated with GFP and Tis11 dsRNA.

Abbr.	CG #	t_1/2_ dsGFP(min ± SD)	t_1/2_ dsTis11(min ± SD)	p(T-test)	n	3′UTR length (nt)	ARE*Score*
**Tis11-sensitive mRNAs**							
Pxn	CG12002	163±62	>240(510±238)	(0.007)	5	180	0
TotA	CG31509	>240	>240		5	153	2.6
Vir-1	CG31764	>240	>240		5	311	4
Lectin-28C	CG7106	>240	>240		5	53	0
CG8239	CG8239	134±90	199±118	0.178	5	287	10.8
NimB2	CG31839	>240	>240		5	81	0
Ric	CG8418	229±95	200±68	0.298	5	1317	10.1
CG15435	CG15435	46±6	60±11	0.018	5	191	11.55
CG7115	CG7115	63±13	85±21	0.040	5	493	6.15
CG10249	CG10249	49±6	44±7	0.131	5	822	6.5
CG2915	CG2915	>240	>240		5	83	0
NimB1	CG33119	>240	>240		5	36	0
**Control mRNAs**							
Reck	CG5392	39±7	35±4	0.161	5	582	11.2
Pax	CG31794	158±56	120±20	0.122	4	325	4.65
CG32512	CG32512	114±43	78±15	0.060	5	583	9.55
Ho	CG14716	77±19	65±11	0.122	5	104	4.1
CG5026	CG5026	183±96	128±42	0.134	5	572	6.5
CG7787	CG7787	>240	168±51		5	438	3.2
Ef1α48D	CG8280	>240	>240		3	554	4.9
CG17184	CG17184	66±11	107±64	0.097	5	653	3.9
CG31997	CG31997	>240	>240		3	70	0
CG10131	CG10131	>240	>240		3	97	0
CG8135	CG8135	>240	170±37		5	148	0
dUTPase	CG4584	>240	>240		3	131	0
Mod(mdg4)	CG32491	93±32	74±14	0.126	5	44	5.2
eEF1δ	CG4912	>240	>240		3	91	0

## Discussion

In this report, we developed ARE*Score* as an algorithm to identify AREs and provide a measure for their potential strength (Figure 1). The ARE*Score* was validated using genome-wide mRNA half-life measurements in human DG75 B-cells [32] and mouse NIH3T3 fibroblast [33]. Although the correlation between ARE*Score* and mRNA half-life was weak (R_S_ = −0.155 and −0.147 in the two data sets, respectively), it was statistically highly significant. To our knowledge, this is the best correlation observed so far between any parameter and mRNA half-lives on a genome-wide scale.

The potential of the ARE*Score* could be further demonstrated with a set of TTP-associated mRNAs that we had previously identified by RNA-IP in mouse macrophages [13]. ARE*Scores* were much higher in this set of mRNAs than in the two control groups (Figure 1H). Among the Tis11-sensitive mRNAs that we identified in *Drosophila* SL2 cells, we also observed an increased frequency of mRNAs with higher ARE*Scores* (Figure 5), suggesting that *Drosophila* Tis11 recognizes AREs with sequence features similar to mammalian AREs.

Khabar *et al.* used bioinformatic tools to generate the ARE-database (ARED), a comprehensive list of potential AREs in the human, mouse and *Drosophila* genome [7], [31], [36]. The principle behind ARED is that it classifies AREs according to the number and density of AUUUA pentamers and surrounding AU-rich sequences, which correlates, to some degree, with the potential strength of the ARE. In contrast to ARED, the purpose of ARE*Score* is not to make categories, but rather generate a single score that provides a measure for the likelihood and potential strength of an ARE. It is important to emphasize that in the absence of experimental validation, neither ARED nor ARE*Score* is able to predict with absolute certainty whether a given mRNA contains a functional ARE. For the ARE*Score*, the false positive rate was visualized by ROC analysis, whereby the ARE*Score* is tested for its ability to discriminate between the 10% most short-lived and the 10% most long-lived mRNAs (Figure 1D and 1G). For ARED, the false positive rate is not known.

An advantage of ARE*Score* is that it can be applied easily to any genome or set of sequences. Thus, we were able to compute the ARE*Score* distribution for the transcriptomes of 14 species representing all but two of the major branches of metazoan evolution (Figure 2 and Figure S1). The analysis showed that mRNAs with high ARE*Scores* are most abundant in man and mouse, the two mammalian species analyzed. Comparison to randomized control sequences revealed that mRNAs with high ARE*Scores* (≥10) are overrepesented in man, mouse, chicken, zebrafish and the fruit fly (Figure 3). This suggests that AREs were under positive selection pressure during the evolution of these organisms. On the other hand, high ARE*Score* mRNAs are underepresented in the sponge *A. queenslandica*, the freshwater cnidarian *H. magnipapillata*, the mollusc *A. californica* and the nematode *C. elegans*, suggesting that AREs did not expand in the genomes of metazoans with simpler body plans. Alternatively, the element corresponding to the ARE might have different sequence features in these organisms.

Given that very little is known about AREs in *D. melanogaster*, we then made use of the ARE*Score* to address the role of AMD in *Drosophila* SL2 cells. Using an FL-based reporter assay, we first tested several factors and found that knocking down Tis11 or Not1 caused inhibition of AMD, whereas the kd of Rox8, AGO1, AGO2, LSm1 or pcm had no effect (Figure 4). The requirement of Tis11 for AMD is in good agreement with the well documented role of TTP in mammalian AMD [37] as well as previous reports demonstrating that Tis11 participates in AMD in *Drosophila* cells [29], [30], [31], [38]. The requirement for Not1 may be linked to our recent finding that mammalian TTP recruits the Caf1 deadenylase through its association with Not1 [23]. Not1 is the scaffold protein of the Ccr4-Caf1-Not deadenylase complex that plays a key role in cytoplasmic mRNA turnover. In *Drosophila*, Not1 was shown to be important for bulk mRNA deadenylation and, more specifically, for the rapid deadenylation of Hsp70 mRNA [34], [35]. A previous report had suggested that AGO1 and AGO2 are required for the rapid degradation of a reporter mRNA containing the ARE of mammalian TNFα in *Drosophila* S2 cells [38]. In our assay, kd of the argonaute proteins AGO1 and AGO2 did not affect expression of the reporter gene containing the ARE of mouse IL-3 (Figure 4A), indicating that AGO proteins are not generally required for AMD.

As potential substrates of AMD, we then identified 53 mRNAs whose expression was elevated after kd of Tis11 (Figure 5A). The ARE*Score* of these Tis11-sensitive mRNAs was found to be higher in comparison to the distribution in the entire *D. melanogaster* transcriptome (Figure 5B), and the difference was statistically significant for mRNAs with ARE*Scores* ≥4 (Table S3). CecA1 mRNA, previously identified as a target of Tis11 [29], [30], [31], did not come up as Tis11-sensitive simply because this mRNA is not represented on the Affymetrix Drosophila Genome 2.0 array that we used for our study.

We then compared the expression levels of 6657 mRNAs in SL2 cells with their ARE*Score* (Figure 6A–6E), and observed that mRNAs with high ARE*Scores* have reduced expression levels. However, this effect may be indirect because the ARE*Score* strongly correlates with 3′UTR length. Indeed, when grouping mRNAs according to their 3′UTR length, we again observed that mRNAs with long 3′UTRs have lower expression levels. The impact of 3′UTR length was in fact stronger than the impact of the ARE*Score*. Long 3′UTRs are likely to correlate with low expression levels through the presence of different suppressive elements including AREs and miRNA-binding sites. Moreover, the distance between the stop codon and the poly(A) tail is a determinant of nonsense-mediated mRNA decay [39] and may thereby as well contribute to mRNA suppression. We also noted that mRNAs with very short 3′UTRs <100 nt are expressed below the overall average. A possible explanation is that very short 3′UTRs might lack stabilizing elements, although there is little experimental evidence that such elements are abundant.

To examine the impact of the ARE*Score* independently of its correlation with 3′UTR length, we chose a group of mRNAs with intermediate 3′UTRs (Figure 6F–6H). Within this group we could observe that mRNAs with an ARE*Score* ≥8 had a more than 3-fold reduced average expression level compared to the mRNAs with ARE*Scores* <8. Since 3′UTR length had a much smaller effect on mRNA levels in this group, we concluded that the ARE*Score* is an independent parameter that correlates with suppressed mRNA levels. Given the multitude of factors that affect mRNA stability and transcription rates, it is remarkable that the ARE*Score* alone has a detectable influence on mRNA expression levels.

Finally, we measured the decay rates of 26 mRNAs in *Drosophila* SL2 cells (Table 1). Indeed, we observed a very strong, negative correlation between mRNA half-life and the ARE*Score* (R_S_ = −0.73, Figure 7), which was higher than the correlation with 3′UTR length (R_S_ = −0.61,). Since we measured mRNA half-lives both in control GFP and Tis11 kd cells, we could also identify three mRNAs that are significantly stabilized by the absence of Tis11. These mRNAs encode for peroxidasin (Pxn), CG15435, a C2H2 zinc finger protein of unknown function, and CG7115, a protein phosphatase of the PP2C family. Taken together, our analysis provides compelling evidence that AREs are functional regulatory elements in *D. melanogaster* cells whose suppressive effect can be detected on a transcriptome-wide level. Interestingly, we found two short-lived mRNAs with a high ARE*Score* (Reck and CG32512) in our control group of mRNAs that are not sensitive to Tis11 kd. This indicates that in addition to Tis11, other proteins also participate in regulating AMD. It is clear that we have only begun to understand the posttranscriptional regulatory network that controls gene expression through mRNA turnover in *D. melanogaster*.

## Methods

### Plasmid construction

Plasmid pRp128-RL (p2933) [40] contains the Drosophila RNA polymerase III 128 kDa subunit promoter to drive RL expression, and was kindly provided by Michael Boutros (German Cancer Research Center, Heidelberg). For pRp128-FL (p2934), pRp128-RL was digested with SpeI/NheI and religated to remove part of the polylinker. In the resulting construct, the RL-containing HindIII/XbaI fragment was replaced with the FL-containing HindIII/XbaI insert from pGL3-Basic (Promega). For pRp128-FL-mIL3-ARE (p2935), the mouse IL-3 ARE sequence (NM_010556.4, nt 680–744) was amplified by PCR using primers G1090/G1091 (Table S4) and inserted into the XbaI site of pRp128-FL. The control vector pRp128-FL-mIL3-INV (p2936) was constructed in the same way with the IL-3 ARE inserted in the opposite orientation.

To generate pAc5-FL-mIL3-ARE (p2937), a 3.8 kb FL-containing HindIII (blunt)–BglII fragment was excised from plasmid pRp128-mIL3-ARE and ligated to KpnI (blunt) - BglII fragment (2.4 kb) with Ac5 promoter obtained by digestion of pAc5.1b-EGFP-dmDcp1 (p2450) (kindly provided by Elisa Izaurralde, Max Planck Institute for Developmental Biology, Tübingen, Germany). For pAc5-FL-Vir1-ARE (p2938), the *D. melanogaster* Vir1 3′UTR (NM_165011.2, nt 1521–1830) was first amplified by RT-PCR using primers G1673/G1674. An ARE-containing 191 nt long fragment (NM_165011.2, nt 1640–1830) was re-amplified by PCR using XbaI site-containing primers G1681/G1679 and inserted into the XbaI site of pRp128-FL to generate pRp128-FL-Vir1-ARE. Finally, the Ac5 promoter was excised as a SapI–BglI fragment from pAc5-FL-mIL3-ARE and cloned into the SapI/BglI sites of pRp128-FL-Vir1-ARE, thereby replacing the pRp128 promoter. pAc5-FL (p2939) was generated in a similar manner by cloning the SapI–BglI fragment from pAc5-FL-mIL3-ARE into the SapI/BglI sites of pRp128-FL.

### Cell culture and transfection

*Drosophila* SL2 cells were cultivated at 26°C under atmospheric CO_2_ in Schneider's Drosophila Medium (Invitrogen-Gibco, Cat. No. 11720-034) supplemented with 10% foetal bovine serum (Biochrome Superior FBS, Cat. No. S0615), 50 U/ml penicillin and 0.05 mg/ml streptomycin (both Pan Biotech).

All DNA transfections were performed using Effectene reagent (Qiagen, Cat. No. 301425) according to the manufacturer's instructions. When combined with RNAi, cells were first treated with dsRNA for two days, followed by DNA transfection for two additional days. For luciferase assays, 10.000 cells were seeded per well of a 384-well plate (Greiner), treated with 250 ng of dsRNA and transfected with 7 ng of FL-encoding and 3 ng of RL-encoding plasmids. Where indicated, ActinomycinD (Applichem, Cat. No. A1489) was used at a concentration of 5 µg/ml. Unless noted otherwise, cell lysis and RNA extraction were performed with Genematrix universal DNA/RNA/Protein purification kit (Eurx), according to manufacturer's instructions.

### dsRNA preparation and treatment

DNA templates for *in vitro* transcription were amplified by PCR using primers containing Sp6 promoter sequences, as specified in Table S5. DNA templates were gel-purified using a gel extraction kit (QIAGEN, Cat. No. 28706). *In vitro* transcription reactions were assembled in a total volume of 50 µl containing 50–75 ng/µl DNA template, 3 mM NTPs each (Promega), 40 U RNasin (Promega, N2111), 0.5 U yeast pyrophospatase (Sigma, Cat. No. I1891), 200 U Sp6 RNA polymerase (Fermentas, EP0133), 80 mM HEPES-KOH pH 7.5, 32 mM MgCl_2_, 2 mM spermidine and 40 mM DTT. Reactions were incubated for 4 hours at 37°C. DNA was then digested by the addition of 1 U/µl DNase RQ1 (Promega) for 15 minutes at 37°C. The synthesized RNA was purified by gel filtration using pre-packed Sephadex G-50 columns (Roche, Cat. No. 11274015001). Strands were annealed by heating the purified RNA to 65°C and allowing it to slowly cool to room temperature. For RNAi, *Drosophila* cells were grown in 6 cm-dishes and incubated with 50 µg of dsRNA per 4 ml of medium for a minimum of 4 days.

### Nothern blot analysis

Total RNA was extracted using the Genematrix universal RNA purification kit (Eurx). 5–12 µg of RNA was resolved by 1.1% agarose/2% formaldehyde/MOPS (morpho-linepropanesulfonic acid) gel electrophoresis and blotted over night with 8× saline-sodium citrate (SSC, 1× contains 0.15 M NaCl and 0.015 M sodium citrate) buffer onto Hybond-N+ Nylon membranes (Amersham, GE Healthcare). Membranes were hybridized overnight at 55°C with digoxigenin-labelled RNA probes synthesized *in vitro* using Sp6 polymerase (Fermentas) and DIG RNA labelling mix (Roche). 500 ng RNA probe was diluted in 10 ml hybridization buffer containing 50% formamide, 5× SSC, 5× Denhard's solution, 5 mM EDTA, 10 mM PIPES pH 7.0, 4 mg torula yeast RNA (US Biological) and 1% SDS. Membranes were washed twice with 2× SSC/0.1% SDS for 5 minutes, and twice with 0.5× SSC/0.1% SDS for 20 minutes at 65°C. Alkaline phosphatase-coupled anti-digoxigenin Fab fragments and CDP-Star substrate (both Roche) were used for detection according to the manufacturer's instructions. Sequences of primers that were used to generate templates for digoxigenin-labelled RNA probes are provided in Table S6.

### Quantitative PCR

For qPCR, total RNA was extracted by Genematrix RNA purification kit (Eurx) and subjected to DNase treatment using RQI DNase (Promega, 1.5 U/column). cDNA was synthesized from 5 µg of total RNA using oligo-dT18 (Invitrogen) and M-MLV H(-) reverse transcriptase (Promega). 1∶40 volume of a cDNA reaction was used for PCR. PCR reactions were assembled in 384-well plates, 15 µl/well final volume. DNA SYBR Green I Master kit (Roche Cat. No. 04707516001) was used according to manufacturer's instructions. Quantitative PCR was performed with the Lightcycler 480 system (Roche). Gene-specific primers sequences are given in Table S7.

### Luciferase assay

FL and RL activities were measured using the Dual Luciferase Reporter Assay system (Promega) or reagents developed in the lab of Michael Boutros (German Cancer Research Centre, DKFZ, Heidelberg). Chemiluminescence was measured using a Mithras LB940 plate reader.

### Genome-wide expression profiling and bioinformatic analysis

*Drosophila* SL2 were treated with either dsRNA-GFP or dsRNA-Tis11 for 4 days. Total RNA was prepared using RNEasy kit from Qiagen (Cat. No. 74106). Efficiency of Tis11 knockdown was confirmed separately by Northern blot and qPCR analyses. Expression profiling was carried out on GeneChip Drosophila Genome 2.0 Arrays (Affymetrix, Cat. No. 900531) at European Molecular Biology Lab Genecore facility (Heidelberg, Germany). Microarray data were deposited at NCBI GEO, accession GSE28147. The RMA algorithm was used for normalization of raw data (RMAExpress software, http://rmaexpress.bmbolstad.com/). Further statistical analyses were performed in the multi-experiment viewer of the TM4 microarray software suite [41] or with R software (http://www.r-project.org). Beside standard Student t-test, we also used microarray-oriented Rank products test [42] to identify significant changes in expression.

### ARE*Score*

The ARE*Score* algorithm was written in Perl and is accessible online at http://arescore.dkfz.de/arescore.pl. ARE*Score* uses either a set of sequences provided in FASTA format or retrieves the 3′UTR sequences of properly annotated transcripts if Refseq IDs are entered. The algorithm first generates a basal score by adding a fixed value of 1 for each AUUUA pentamer identified. It then calculates the distance between neighboring pentamers, and adds a value to the basal score if pentamers are in close proximity. A value is also added when pentamers are located within a region of high AU content, termed an AU-block in ARE*Score*. In its standard setting, ARE*Score* adds a value of 1.5 for overlapping pentamers, 0.75 if pentamers are 0–3 nt apart, 0.4 if pentamers are 4–6 nt apart, 0.2 if pentamers are 7–9 nt apart, and 0.3 if pentamers are within an AU-block. By default, an AU-block starts when a sequence of 20 nt (word size) has an AU content of ≥80%. The block ends when the AU content drops below 55% within the chosen word size. To increase the flexibility of ARE*Score*, users can change the values that are added to the basal score, and alter the settings that define an AU-block.

For the transcriptome-wide ARE*Score* analysis, transcripts with properly formated feature fields (Genbank GeneID and Accession identifiers) were downloaded from Refseq, and the ARE*Score* was determined for every 3′UTR ≥10 nt in length. If several transcripts map to the same gene locus (identical GeneIDs), only the mRNA with the highest ARE*Score* was taken into the analysis. To generate a fully matched set of randomized control sequences, nucleotides were randomly chosen from the pool of all 3′UTRs analyzed, and assembled into sequences identical in length to the original 3′UTRs. The ARE*Score* was then determined for the randomized control sequences as well.

## Supporting Information

Figure S1 AREScore distribution in comparison to randomized controls. The AREScore was calculated for all annotated 3′UTRs ≥10 nt in legth in the transcriptomes of 14 metazoan species. The AREScore distribution was compared to fully adjusted, randomized control sets of sequences whose lengths and A/T/G/C-content were identical to the 3′UTRs of the respective transcriptome. Percentage of transcripts were depicted on a logarithmic scale for the following species: (A) *Homo sapiens*, man; (B) *Mus musculus*, common house mouse; (C) *Gallus gallus*, chicken; (D) *Xenopus laevis*, African clawed frog; (E) *Danio rerio*, zebrafish; (F) *Branchiostoma floridae*, Florida lancelet; (G) *Ciona intestinalis*, vase tunicate; (H) *Strongylocentrotus purpuratus*, purple sea urchin; (I) *Drosophila melanogaster*, fruit fly; (J) *Ixodes scapularis*, deer tick; (K) *Caenorhabditis elegans*, roundworm; (L) *Aplysia californica*, California sea hare; (M) *Hydra magnipapillata*, freshwater polyp; (N) *Amphimedon queenslandica*, demosponge.
(PDF)

Figure S2 Reporter gene expression. (A) Schematic representation of reporter genes containing the *Drosophila* RpIII128 promoter, the coding sequence of firefly luciferase (FL) and an SV40 3′UTR including a polyadenylation signal. The 64 nt long ARE of mouse IL-3 was inserted at the beginning of the 3′UTR. The inverse (INV) sequence of the ARE was used as a negative control. (B) SL2 cells were treated with water or 12.5 µg/ml dsRNAs against GFP or Tis11 over a period of 4 days. On day 3, cells were transiently transfected with pRp128-FL, pRp128-FL-mIL3-ARE or pRp128-FL-mIL3-INV together with pRp128-RL. FL activities were measured on day 5 and normalized to RL. The graph shows average FL/RL ratios ± SE based on 3 biological repeats. (C) SL2 cells were transiently transfected with pRp128-FL, pRp128-FL-mIL3-ARE or pRp128-FL-mIL3-INV. After 48 hours, total RNA was extracted and 8 µg per sample were subjected to Northern Blot analysis using a probe against FL. The endogenous mRNA encoding ribosomal protein RpS20 serves as a loading control. (D) The degradation of FL, FL-mIL3-ARE and FL-mIL3-INV mRNA was determined in transiently transfected SL2 cells. Following treatment with actinomycin D (5 µg/ml), total RNA was extracted at one hour intervals and analyzed by Northern blotting. mRNA signal intensities were quantified, and the FL/RpS20 mRNA ratio was plotted against time in the bottom panel.
(PDF)

Figure S3 Sequence of the mouse IL3 ARE and the *Drosophila* Vir1 ARE. Depicted are the sequences that were inserted into the FL-mIL3-ARE and FL-Vir1-ARE reporter genes. The mIL3-ARE sequence corresponds to a 65 nt long fragment derived from *M. musculus* NM_010556.4 (nt 680–744); the Vir1-ARE sequence corresponds to a 191 nt long fragment derived from *D. melanogaster* NM_165011.2 (nt 1640–1830).
(PDF)

Figure S4 Confirmation of Tis11-dependent mRNA expression. (A) The expression levels of 20 Tis11-sensitive mRNAs were measured by qPCR in SL2 cells after treatment for 4 days with dsRNAs against Tis11 or GFP. Expression levels were normalized to RpS20 mRNA, and plotted as the ratio between the normalized level in Tis11 kd cells and the normalized level in GFP kd cells (green bars). The fold change observed in the microarray analysis is represented by red bars. (B) The same analysis was carried out for 15 control mRNAs, whose expression was not affected by the knock down of Tis11.
(PDF)

Figure S5 Degradation rates of Tis11-sensitive and control mRNAs. (A) The decay rates of 12 Tis11-sensitive mRNAs were measured in SL2 cells upon kd of Tis11 or, as a control, GFP. After treating cells with the corresponding dsRNAs for four days, Actinomycin D (5 µg/ml) was added, and total RNA was extracted 0, 30, 60 and 120 minutes later. mRNA levels were measured by qPCR, normalized to RpS20 mRNA, and represented as % of the initial value at time point 0. Shown are average values ± SD from 3–5 repeat experiments. Half-lives are listed in Table 1. (B) The decay rates of 15 control mRNAs, whose expression is not affected by Tis11 kd, were measured by qPCR in SL2 cells as described above. Shown are average values ± SD from 3–5 repeat experiments.
(PDF)

Table S1 Comparison of ARE*Score* between TTP-associated mRNAs and mouse transcriptome.
(PDF)

Table S2 Comparison of ARE*Score* between TTP-associated mRNAs and concatemer control sequences.
(PDF)

Table S3 Comparison of ARE*Score* between Tis11-sensitive mRNAs and *D. melanogaster* transcriptome.
(PDF)

Table S4 Oligonucleotides used for reporter plasmid cloning.
(PDF)

Table S5 Oligonucleotides used for dsRNA synthesis templates.
(PDF)

Table S6 Oligonucleotides used for Northern blot probes.
(PDF)

Table S7 Oligonucleotides used for qPCR.
(PDF)
