# Supplementary material for: Genome-Wide Assessment of AU-Rich Elements by the AREScore Algorithm
Source: PLoS Genet. 2012 Jan 5;8(1):e1002433. doi: 10.1371/journal.pgen.1002433 (PMC3252268; doi:10.1371/journal.pgen.1002433)
Supplement: Figure S1 — AREScore distribution in comparison to randomized controls. The AREScore was calculated for all annotated 3′UTRs ≥10 nt in legth in the transcriptomes of 14 metazoan species. The AREScore distribution was compared to fully adjusted, randomized control sets of sequences whose lengths and A/T/G/C-content were identical to the 3′UTRs of the respective transcriptome. Percentage of transcripts were depicted on a logarithmic scale for the following species: (A) Homo sapiens, man; (B) Mus musculus, common house mouse; (C) Gallus gallus, chicken; (D) Xenopus laevis, African clawed frog; (E) Danio rerio, zebrafish; (F) Branchiostoma floridae, Florida lancelet; (G) Ciona intestinalis, vase tunicate; (H) Strongylocentrotus purpuratus, purple sea urchin; (I) Drosophila melanogaster, fruit fly; (J) Ixodes scapularis, deer tick; (K) Caenorhabditis elegans, roundworm; (L) Aplysia californica, California sea hare; (M) Hydra magnipapillata, freshwater polyp; (N) Amphimedon queenslandica, demosponge. (PDF) [file pgen.1002433.s001.pdf]

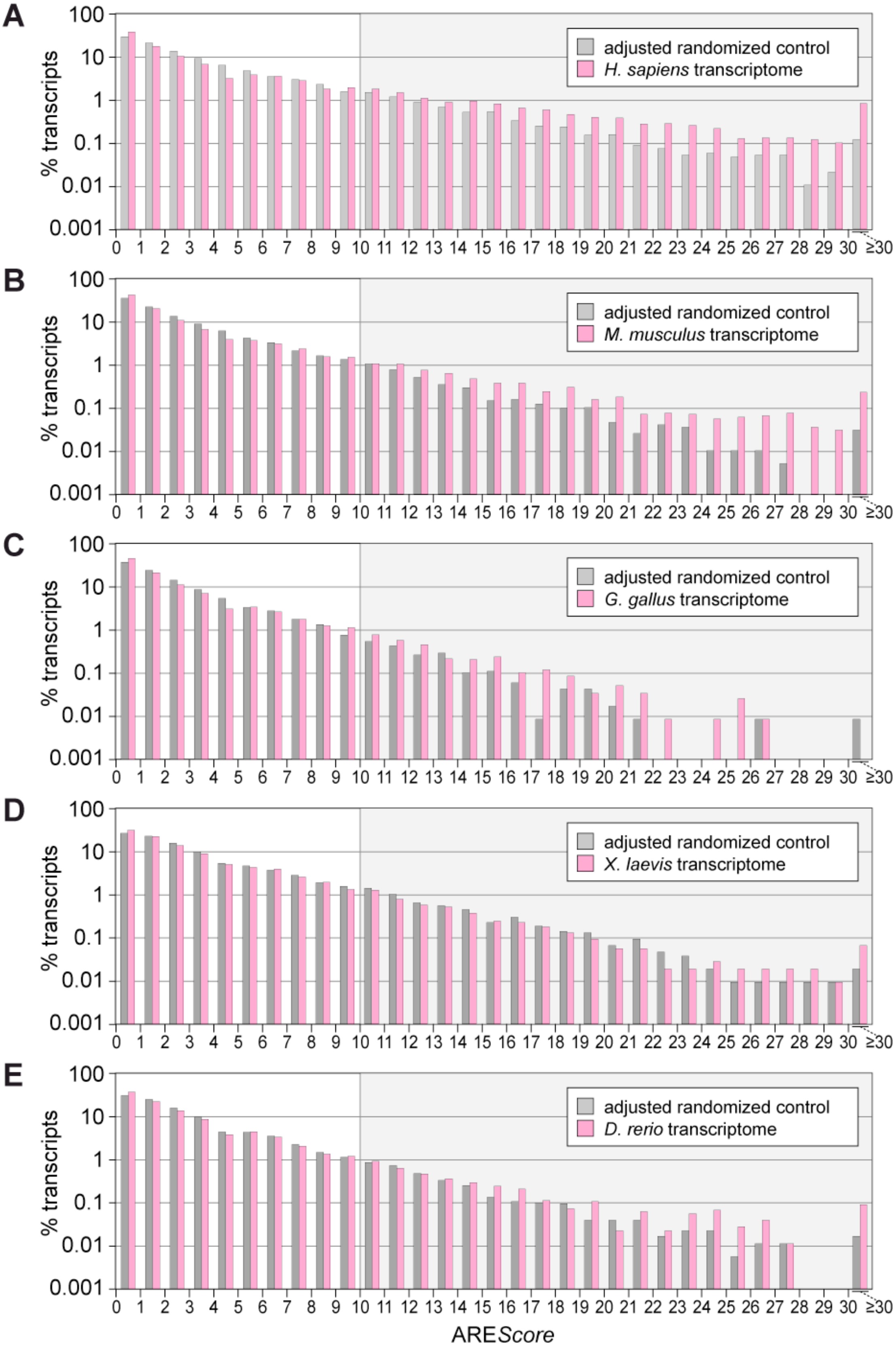
A
B
C
D
E
% transcripts
100
10
1
0.1
0.01
0.001
0 1 2 3 4 5 6 7 8 9 10 11 12 13 14 15 16 17 18 19 20 21 22 23 24 25 26 27 28 29 30 ≥30
adjusted randomized control
H. sapiens transcriptome
M. musculus transcriptome
G. gallus transcriptome
X. laevis transcriptome
D. rerio transcriptome
ARE*Score*

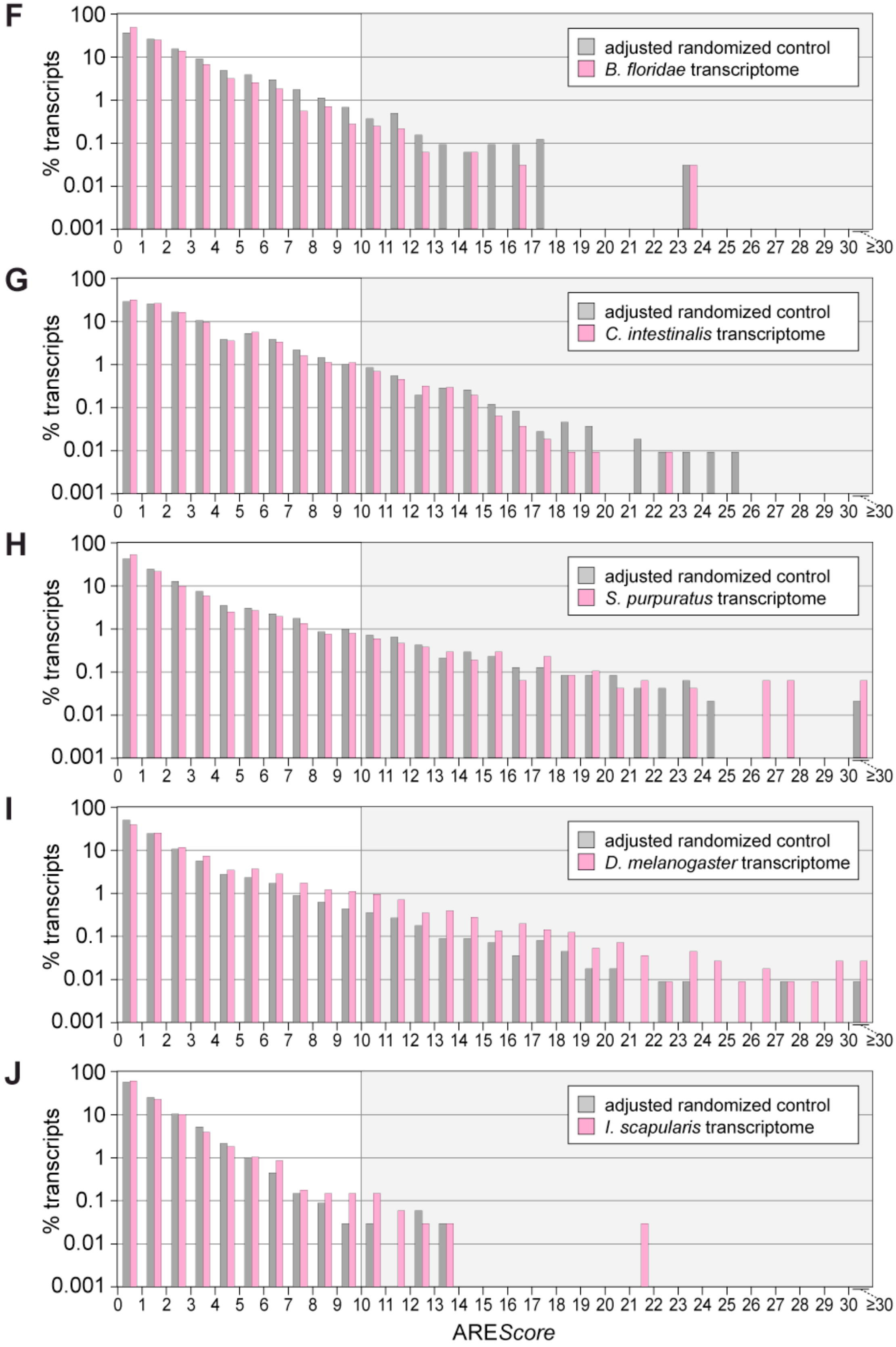
F
G
H
I
J
% transcripts
100
10
1
0.1
0.01
0.001
0 1 2 3 4 5 6 7 8 9 10 11 12 13 14 15 16 17 18 19 20 21 22 23 24 25 26 27 28 29 30 ≥30
adjusted randomized control
B. floridae transcriptome
C. intestinalis transcriptome
S. purpuratus transcriptome
D. melanogaster transcriptome
I. scapularis transcriptome
AREScore

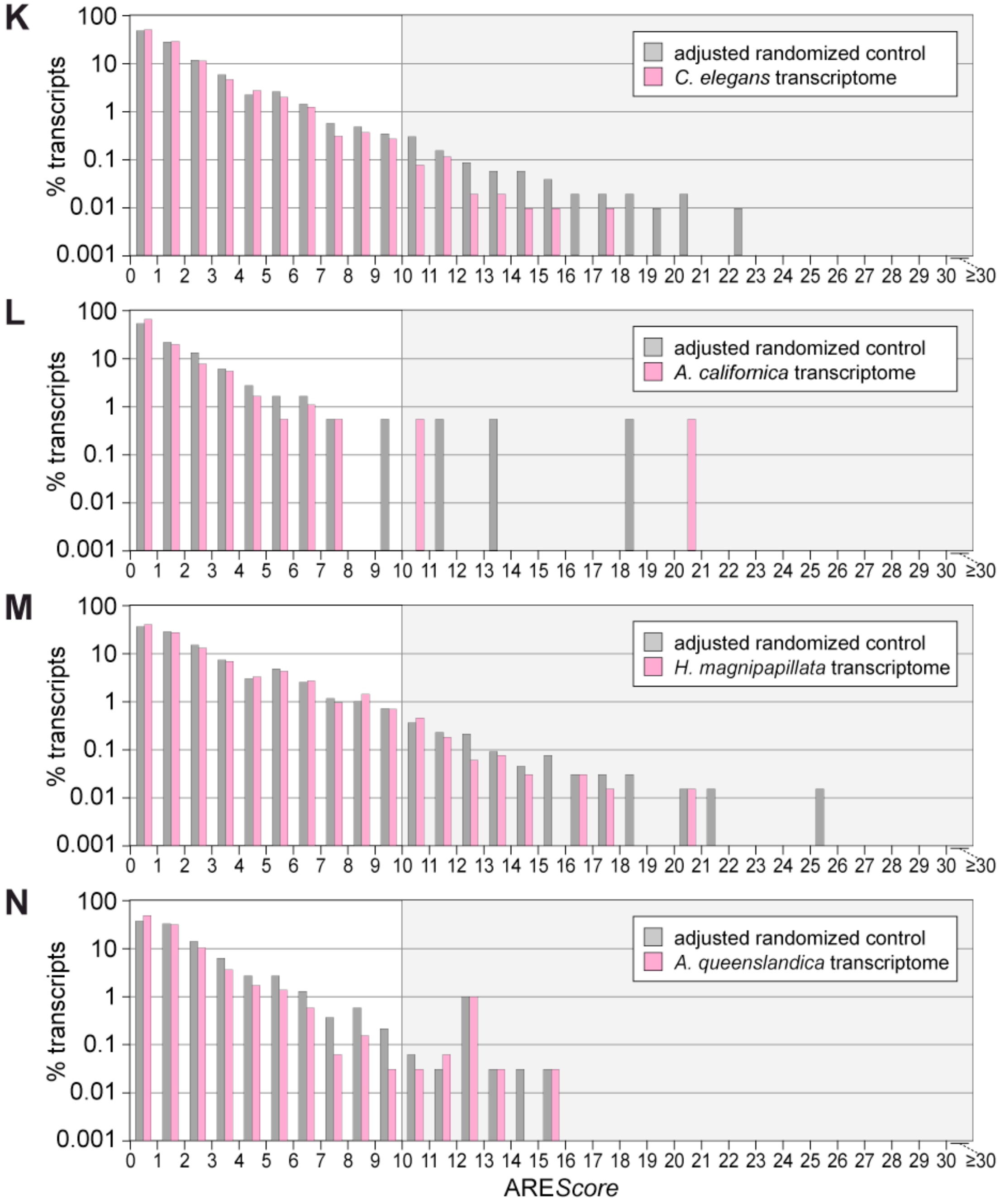

Figure S1. ARE*Score* distribution in comparison to randomized controls. The ARE*Score* was calculated for all annotated 3'UTRs ≥10 nt in legth in the transcriptomes of 14 metazoan species. The ARE*Score* distribution was compared to fully adjusted, randomized control sets of sequences whose lengths and A/T/G/C-content were identical to the 3'UTRs of the respective transcriptome. Percentage of transcripts were depicted on a logarithmic scale for the following species: (A) *Homo sapiens*, man; (B) *Mus musculus*, common house mouse; (C) *Gallus gallus*, chicken; (D) *Xenopus laevis*, African clawed frog; (E) *Danio rerio*, zebrafish; (F) *Branchiostoma floridae*, Florida lancelet; (G) *Ciona intestinalis*, vase tunicate; (H) *Strongylocentrotus purpuratus*, purple sea urchin; (I) *Drosophila melanogaster* , fruit fly; (J) *Ixodes scapularis*, deer tick; (K) *Caenorhabditis elegans*, roundworm; (L) *Aplysia californica*, California sea hare; (M) *Hydra magnipapillata*, freshwater polyp; (N) *Amphimedon queenslandica*, demosponge.
