# Supplementary material for: Genome-Wide Assessment of AU-Rich Elements by the AREScore Algorithm
Source: PLoS Genet. 2012 Jan 5;8(1):e1002433. doi: 10.1371/journal.pgen.1002433 (PMC3252268; doi:10.1371/journal.pgen.1002433)
Supplement: Figure S2 — Reporter gene expression. (A) Schematic representation of reporter genes containing the Drosophila RpIII128 promoter, the coding sequence of firefly luciferase (FL) and an SV40 3′UTR including a polyadenylation signal. The 64 nt long ARE of mouse IL-3 was inserted at the beginning of the 3′UTR. The inverse (INV) sequence of the ARE was used as a negative control. (B) SL2 cells were treated with water or 12.5 µg/ml dsRNAs against GFP or Tis11 over a period of 4 days. On day 3, cells were transiently transfected with pRp128-FL, pRp128-FL-mIL3-ARE or pRp128-FL-mIL3-INV together with pRp128-RL. FL activities were measured on day 5 and normalized to RL. The graph shows average FL/RL ratios ± SE based on 3 biological repeats. (C) SL2 cells were transiently transfected with pRp128-FL, pRp128-FL-mIL3-ARE or pRp128-FL-mIL3-INV. After 48 hours, total RNA was extracted and 8 µg per sample were subjected to Northern Blot analysis using a probe against FL. The endogenous mRNA encoding ribosomal protein RpS20 serves as a loading control. (D) The degradation of FL, FL-mIL3-ARE and FL-mIL3-INV mRNA was determined in transiently transfected SL2 cells. Following treatment with actinomycin D (5 µg/ml), total RNA was extracted at one hour intervals and analyzed by Northern blotting. mRNA signal intensities were quantified, and the FL/RpS20 mRNA ratio was plotted against time in the bottom panel. (PDF) [file pgen.1002433.s002.pdf]

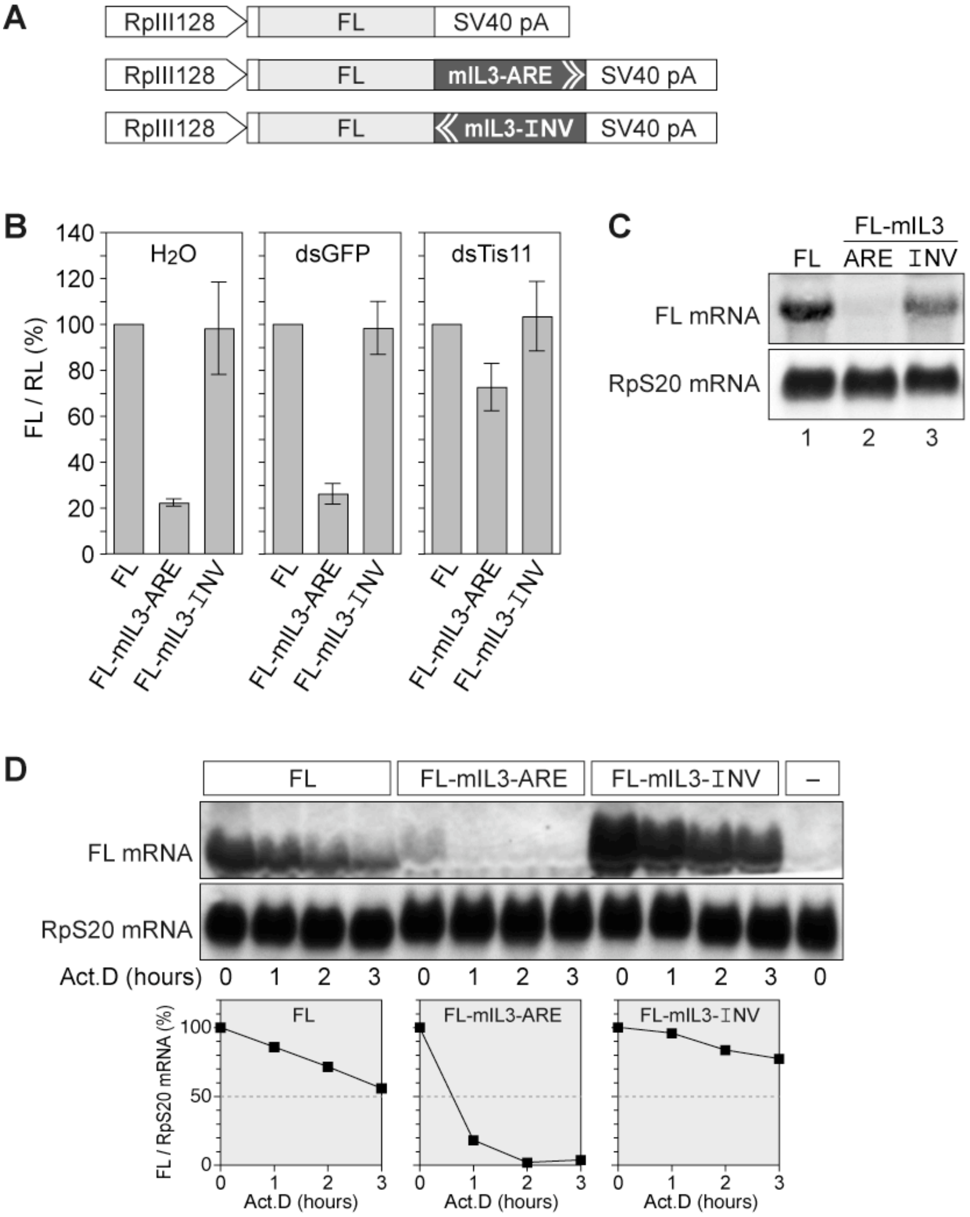

Figure S2. Reporter gene expression. (A) Schematic representation of reporter genes containing the *Drosophila* RpIII128 promoter, the coding sequence of firefly luciferase (FL) and an SV40 3'UTR including a polyadenylation signal. The 64 nt long ARE of mouse IL-3 was inserted at the beginning of the 3'UTR. The inverse (INV) sequence of the ARE was used as a negative control. (B) SL2 cells were treated with water or 12.5 µg/ml dsRNAs against GFP or Tis11 over a period of 4 days. On day 3, cells were transiently transfected with pRp128-FL, pRp128-FL-mIL3-ARE or pRp128-FL-mIL3-INV together with pRp128-RL. FL activities were measured on day 5 and normalized to RL. The graph shows average FL / RL ratios ± SE based on 3 biological repeats. (C) SL2 cells were transiently transfected with pRp128-FL, pRp128-FL-mIL3-ARE or pRp128-FL-mIL3-INV. After 48 hours, total RNA was extracted and 8 µg per sample were subjected to Northern Blot analysis using a probe against FL. The endogenous mRNA encoding ribosomal protein RpS20 serves as a loading control. (D) The degradation of FL, FL-mIL3-ARE and FL-mIL3-INV mRNA was determined in transiently transfected SL2 cells. Following treatment with actinomycin D (5 µg/ml), total RNA was extracted at one hour intervals and analyzed by Northern blotting. mRNA signal intensities were quantified, and the FL / RpS20 mRNA ratio was plotted against time in the bottom panel.
