# Supplementary material for: Genome-Wide Assessment of AU-Rich Elements by the AREScore Algorithm
Source: PLoS Genet. 2012 Jan 5;8(1):e1002433. doi: 10.1371/journal.pgen.1002433 (PMC3252268; doi:10.1371/journal.pgen.1002433)
Supplement: Figure S3 — Sequence of the mouse IL3 ARE and the Drosophila Vir1 ARE. Depicted are the sequences that were inserted into the FL-mIL3-ARE and FL-Vir1-ARE reporter genes. The mIL3-ARE sequence corresponds to a 65 nt long fragment derived from M. musculus NM_010556.4 (nt 680–744); the Vir1-ARE sequence corresponds to a 191 nt long fragment derived from D. melanogaster NM_165011.2 (nt 1640–1830). (PDF) [file pgen.1002433.s003.pdf]

>mIL3-ARE

ATTTTATTCCATTAAGGCTATTTATTTATGTATTTATGTATTTATTTATT
TATTGCCTTCTGTGA

>Vir1-ARE

TAATTTATCCTATTTATTTTCACAGACAACGAGTATTATGTATGCAAGGT
GACATTGAAAATTATTTATTGTTAAAATAAAAGAAATCAAAAATAAGTGT
ATTAATATAAATAAGTCTTCGATAATTGGCTTAATTTATAATGAAGTGCA
ATAAAATATATGCGATGTCTTTGATAAAACTGACTATTATA

Figure S3. Sequence of the mouse IL3 ARE and the *Drosophila* Vir1 ARE. Depicted are the sequences that were inserted into the FL-mIL3-ARE and FL-Vir1-ARE reporter genes. The mIL3-ARE sequence corresponds to a 65 nt long fragment derived from *M. musculus* NM_010556.4 (nt 680–744); the Vir1-ARE sequence corresponds to a 191 nt long fragment derived from *D. melanogaster* NM_165011.2 (nt 1640–1830).
