# Supplementary material for: Genome-Wide Assessment of AU-Rich Elements by the AREScore Algorithm
Source: PLoS Genet. 2012 Jan 5;8(1):e1002433. doi: 10.1371/journal.pgen.1002433 (PMC3252268; doi:10.1371/journal.pgen.1002433)
Supplement: Figure S4 — Confirmation of Tis11-dependent mRNA expression. (A) The expression levels of 20 Tis11-sensitive mRNAs were measured by qPCR in SL2 cells after treatment for 4 days with dsRNAs against Tis11 or GFP. Expression levels were normalized to RpS20 mRNA, and plotted as the ratio between the normalized level in Tis11 kd cells and the normalized level in GFP kd cells (green bars). The fold change observed in the microarray analysis is represented by red bars. (B) The same analysis was carried out for 15 control mRNAs, whose expression was not affected by the knock down of Tis11. (PDF) [file pgen.1002433.s004.pdf]

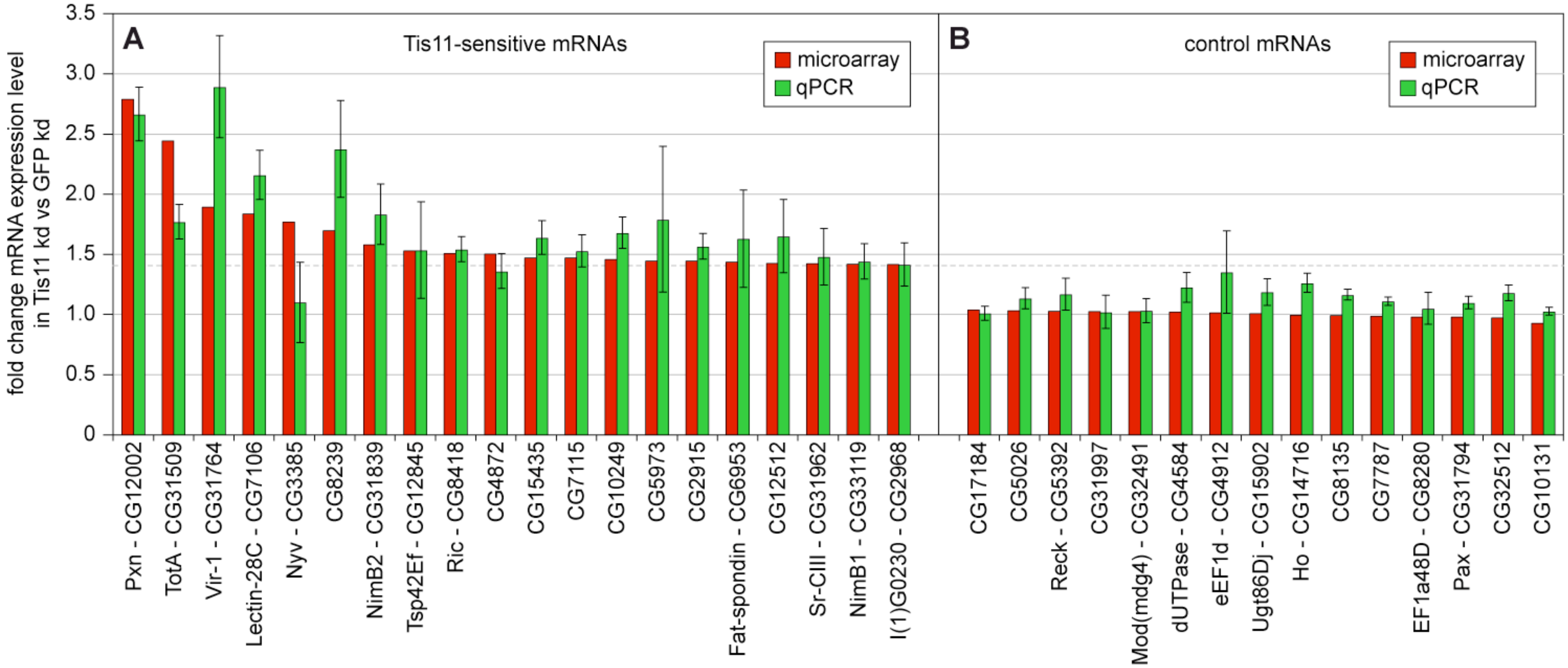

Figure S4. Confirmation of Tis11-dependent mRNA expression. (A) The expression levels of 20 Tis11-sensitive mRNAs were measured by qPCR in SL2 cells after treatment for 4 days with dsRNAs against Tis11 or GFP. Expression levels were normalized to RpS20 mRNA, and plotted as the ratio between the normalized level in Tis11 kd cells and the normalized level in GFP kd cells (green bars). The fold change observed in the microarray analysis is represented by red bars. (B) The same analysis was carried out for 15 control mRNAs, whose expression was not affected by the knock down of Tis11.
