# Supplementary material for: Genome-Wide Assessment of AU-Rich Elements by the AREScore Algorithm
Source: PLoS Genet. 2012 Jan 5;8(1):e1002433. doi: 10.1371/journal.pgen.1002433 (PMC3252268; doi:10.1371/journal.pgen.1002433)
Supplement: Figure S5 — Degradation rates of Tis11-sensitive and control mRNAs. (A) The decay rates of 12 Tis11-sensitive mRNAs were measured in SL2 cells upon kd of Tis11 or, as a control, GFP. After treating cells with the corresponding dsRNAs for four days, Actinomycin D (5 µg/ml) was added, and total RNA was extracted 0, 30, 60 and 120 minutes later. mRNA levels were measured by qPCR, normalized to RpS20 mRNA, and represented as % of the initial value at time point 0. Shown are average values ± SD from 3–5 repeat experiments. Half-lives are listed in Table 1. (B) The decay rates of 15 control mRNAs, whose expression is not affected by Tis11 kd, were measured by qPCR in SL2 cells as described above. Shown are average values ± SD from 3–5 repeat experiments. (PDF) [file pgen.1002433.s005.pdf]

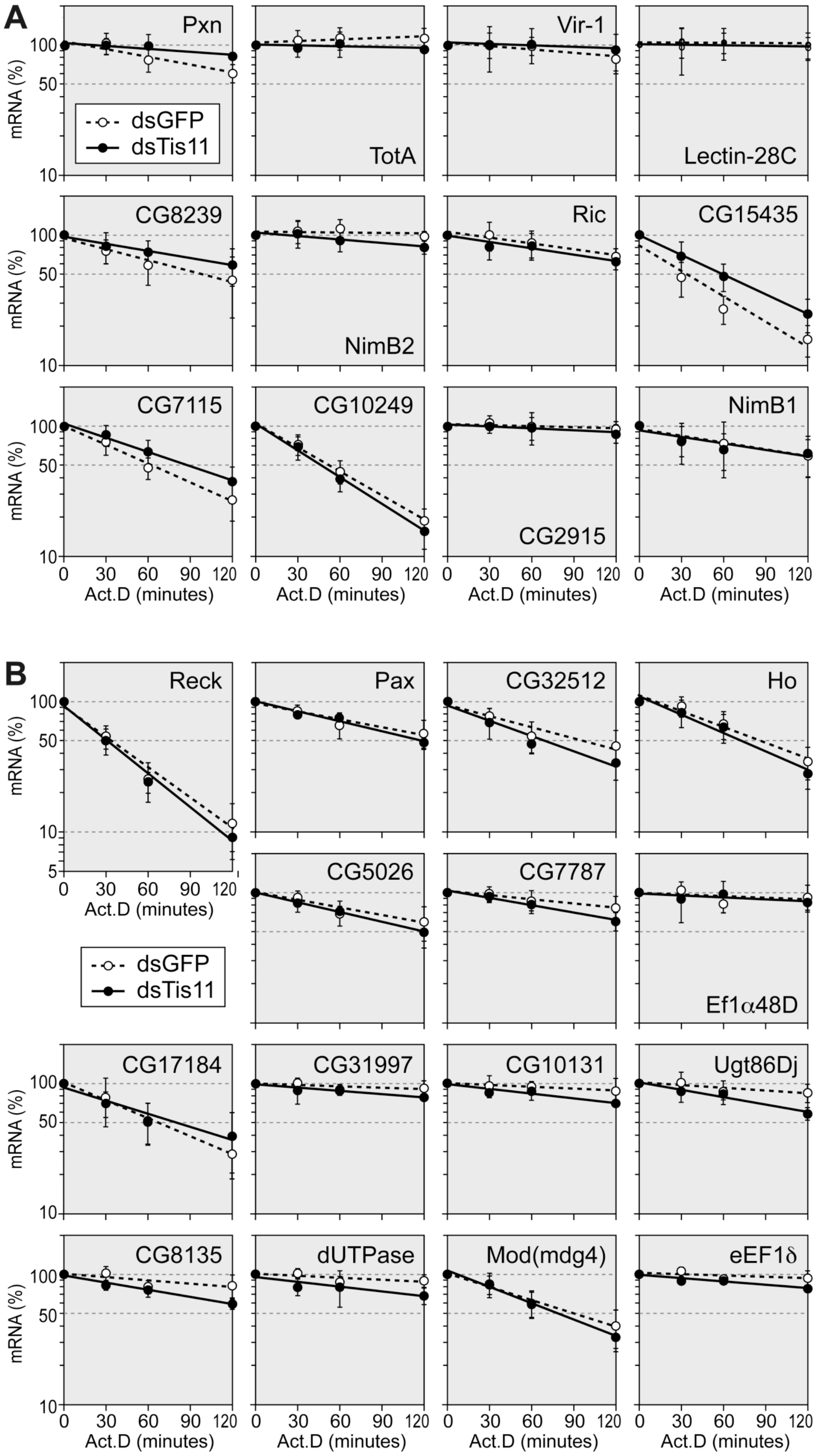

Figure S5. Degradation rates of Tis11-sensitive and control mRNAs. (A) The decay rates of 12 Tis11-sensitive mRNAs were measured in SL2 cells upon kd of Tis11 or, as a control, GFP. After treating cells with the corresponding dsRNAs for four days, Actinomycin D (5 μg/ml) was added, and total RNA was extracted 0, 30, 60 and 120 minutes later. mRNA levels were measured by qPCR, normalized to RpS20 mRNA, and represented as % of the initial value at time point 0. Shown are average values ± SD from 3–5 repeat experiments. Half-lives are listed in Table 1. (B) The decay rates of 15 control mRNAs, whose expression is not affected by Tis11 kd, were measured by qPCR in SL2 cells as described above. Shown are average values ± SD from 3–5 repeat experiments.
