# Supplementary material for: Genome-Wide Assessment of AU-Rich Elements by the AREScore Algorithm
Source: PLoS Genet. 2012 Jan 5;8(1):e1002433. doi: 10.1371/journal.pgen.1002433 (PMC3252268; doi:10.1371/journal.pgen.1002433)
Supplement: Table S1 — Comparison of AREScore between TTP-associated mRNAs and mouse transcriptome. (PDF) [file pgen.1002433.s006.pdf]

Table S1. Comparison of ARE*Score* between TTP-associated mRNAs and mouse transcriptome

	ARE*Score*		
	<4	≥4	
Mouse transcriptome	15461	4372	19833
TTP-associated mRNAs	31	104	135
	15492	4476	19968
χ^2-test	$p < 0.0001$		
