# Supplementary material for: Genome-Wide Assessment of AU-Rich Elements by the AREScore Algorithm
Source: PLoS Genet. 2012 Jan 5;8(1):e1002433. doi: 10.1371/journal.pgen.1002433 (PMC3252268; doi:10.1371/journal.pgen.1002433)
Supplement: Table S3 — Comparison of AREScore between Tis11-sensitive mRNAs and D. melanogaster transcriptome. (PDF) [file pgen.1002433.s008.pdf]

**Table S3.** Comparison of ARE*Score* between Tis11-sensitive mRNAs and *D. melanogaster* transcriptome

| | ARE*Score* | | |
|---|---|---|---|
| | <4 | ≥4 | |
| *Drosophila* transcriptome | 9359 | 1970 | 11329 |
| Tis11-sensitive mRNAs | 32 | 17 | 49 |
| | 9391 | 1987 | 11378 |
| $\chi^2$-test | p = 0.0011 | | |
