# Supplementary material for: Genome-Wide Assessment of AU-Rich Elements by the AREScore Algorithm
Source: PLoS Genet. 2012 Jan 5;8(1):e1002433. doi: 10.1371/journal.pgen.1002433 (PMC3252268; doi:10.1371/journal.pgen.1002433)
Supplement: Table S4 — Oligonucleotides used for reporter plasmid cloning. (PDF) [file pgen.1002433.s009.pdf]

Table S4. Oligonucleotides used for reporter plasmid cloning

Gene	Oligo	Sequence (5'–3')
mouse IL3	G1090	tgcagtctagaattttattccattaag
	G1091	cttactctagatcacagaaggcaata
D.m. Vir-1	G1673	ggtttctaagtctctcccacta
	G1674	tataatagtcagttttatcaaagacat
	G1681	tgcagtctagataatttatcctatttattttca
	G1679	cttactctagatataatagtcagttt
