# Supplementary material for: Genome-Wide Assessment of AU-Rich Elements by the AREScore Algorithm
Source: PLoS Genet. 2012 Jan 5;8(1):e1002433. doi: 10.1371/journal.pgen.1002433 (PMC3252268; doi:10.1371/journal.pgen.1002433)
Supplement: Table S5 — Oligonucleotides used for dsRNA synthesis templates. (PDF) [file pgen.1002433.s010.pdf]

Table S5. Oligonucleotides used for dsRNA synthesis templates

Gene	Oligo	Sequence (5'–3')
Tis11	G1258	gtcatttaggtgacactatagaatactagccaattctatctgccgc
	G1259	gtcatttaggtgacactatagaatacgccctgaccgatgatcatgc
GFP	G1244	gtcatttaggtgacactatagaatacaccctcgtgaccaccctg
	G1245	gtcatttaggtgacactatagaatacgaccatgtgatcgcgct
Rox8	G1173	gtcatttaggtgacactatagccggtgtaaagggaagtcaa
	G1174	gtcatttaggtgacactatagctgttgctgcgtcatcatct
AGO1	G1398	gtcatttaggtgacactatagaatacggcaatcgatggtttc
	G1399	gtcatttaggtgacactatagaatacatgatggaggtacga
AGO2	G1205	gtcatttaggtgacactatagaatacctggtgactcgaccattg
	G1206	gtcatttaggtgacactatagaatacaacttcatgccatccatg
Lsm1	G1201	gtcatttaggtgacactatagaatactggacgacttaaatccgctggc
	G1202	gtcatttaggtgacactatagaatacttagcagaagtcctcgttgattatg
Pcm	G1203	gtcatttaggtgacactatagaatactgggcgttcccaagttctttcgc
	G1204	gtcatttaggtgacactatagaatacgacccaggtgcagcaggaagaag
Not1	G1582	gtcatttaggtgacactatagaatacccggctaagttgttg
	G1583	gtcatttaggtgacactatagaatactgaagctggaaaagtg
