# Supplementary material for: Genome-Wide Assessment of AU-Rich Elements by the AREScore Algorithm
Source: PLoS Genet. 2012 Jan 5;8(1):e1002433. doi: 10.1371/journal.pgen.1002433 (PMC3252268; doi:10.1371/journal.pgen.1002433)
Supplement: Table S6 — Oligonucleotides used for Northern blot probes. (PDF) [file pgen.1002433.s011.pdf]

Table S6. Oligonucleotides used for Northern blot probes

Gene	Oligo	Sequence (5'–3')
FL	G1094	ttaagtgttgttccattccatcacgg
	G1095	gccgatttaggtgacactatagaatacaatcataggacctc
RpS20	G1073	agatttgaatatctcgaccag
	G1097	gccgatttaggtgacactatagaatactgtgtatggaaaaag
Tis11	G1233	tagccaattctatctgccgc
	G1234	gtcatttaggtgacactatagaatacgactgccacgcgtttagagt
