# Supplementary material for: Genome-Wide Assessment of AU-Rich Elements by the AREScore Algorithm
Source: PLoS Genet. 2012 Jan 5;8(1):e1002433. doi: 10.1371/journal.pgen.1002433 (PMC3252268; doi:10.1371/journal.pgen.1002433)
Supplement: Table S7 — Oligonucleotides used for qPCR. (PDF) [file pgen.1002433.s012.pdf]

Table S7. Oligonucleotides used for qPCR

Gene	Oligo	Sequence (5'–3')
Rpl32/RP49	G1293	atgaccatccgcccagcataca
	G1294	tgcgcttgttcgatccgtaacc
Tis11	G1289	aacaccatccagcatccaactcac
	G1290	cgaagttacgccaccggaaatc
Pxn	G1995	aactgttccaaactgcccatgctg
	G1996	agacattatagccgggcattccgt
TotA	G1686	gcacccaggaactacttgacatct
	G1687	gacctccctgaatcggaactc
Vir-1	G1602	aggtgcgcgacattgatga
	G1603	ggtgcgctggtgtgaaga
Lectin-28C	G1997	agccagaagacatcgctaacggaa
	G1998	tctcgatccgattcgcttgaacct
Nvy	G2293	aggtgaaacgccaagctgtcattg
	G2294	attgacggcgacttgttgtccaga
CG8239	G1647	ctgcagcgctgtttgaatgaa
	G1648	cgatgtgcagcttccaggtt
NimB2	G1943	tgctccgaatacctgtgtctgcat
	G1944	ttgcattccggctggcagtactta
Tsp42Ef	G2291	tgtggcggtgtcctacaacctaaa
	G2292	gcaatgatgaccaggcacaggaaa
Ric	G1923	aactaataacccgtgtccgcctgt
	G1924	aatctcgcggaccaacgtgtagaa
CG4872	G1993	tatgtgggcaactacaggctggaa
	G1994	attcccactgaacgaggtcaccaa
CG15435	G1921	accaagttgctcgagattaggcga
	G1922	attggcggctcagctatcttctca
CG7115	G1975	atacagatagcccggcatttccca
	G1976	cccaccgcaaatttggacaggatt
CG10249	G1971	gccatcgcagtaaagccatttcgt
	G1972	ttccgcagtatggcggtatggaat
CG5973	G1939	gccgccatgttgctggattacatt
	G1940	agccttcgcctcaatatgggagat
CG2915	G1935	tggaattcatgcgagggagtggat
	G1936	tcggtcgtgctcgaatgtgtactt
Fat-spondin	G1985	tcccaaggattatccgttcgccat
	G1986	aaggagaagttggactcgtgcgaa

Table S7 (continued). Oligonucleotides used for qPCR

Gene	Oligo	Sequence (5'–3')
CG12512	G1983	tctgacggatcacatcgaagccaa
	G1984	tcatcatcgtggtagcccagcatt
Sr-CIII	G1989	acacgcttccagaacctaccactt
	G1990	ttccacatatcctcgaccgccatt
NimB1	G1991	gtatgccaatgaatgtgtgccgga
	G1992	atcggacaggtgtgaatacaggca
I(1)G0230	G1987	gccaaatcgatgtgccttccttct
	G1988	aaccgctggagacgaagaacttga
Reck	G2006	tattggtccgcctgtcaatccagt
	G2007	atcgacccacataatccaccagca
Pax	G2016	aaggttagcaacggaacaaacggc
	G2017	aatccaactgatcctcgcgactgt
CG32512	G2008	ccttgtttgcgacgacctttccaa
	G2009	ctaaatgtgctgtggtttgcgggt
Ho	G2018	gcaaagatcgcacttgccctttct
	G2019	tccggcagatgggtctcaaagaat
CG5026	G2010	ttcttgaatccgctgtacgagcct
	G2011	actgtgactgagctgcgttgatct
CG7787	G2020	ttgatgctgaaagcacaagagggc
	G2021	tcatgtccttgaccagccagaagt
EF1a48D	G2014	gggcaagaagtagctggtttgctt
	G2015	tattgtgttcgctgctgctgttgc
CG17184	G2024	tttgctgaacgagcacgctctcta
	G2025	atggttcgccatgcagtgtttctg
CG31997	G2030	gccttcacagtttctctgtgtgct
	G2031	tggcacatcgagttgaagcaaagc
CG10131	G2034	aaagagagattcagggcttcgcca
	G2035	tagcagggcatatcgcaatcccaa
Ugt86Dj	G2036	aactgatcggaatttgcacctgcg
	G2037	gcacgaaatggcattatgggctct
CG8135	G2042	tattgctaccgaactggtgcagga
	G2043	agtaatcgctgcccagaacctgat
dUTPase	G2044	aatctcggcgtcgtcctgttcaat
	G2045	tgcggatagaagatacgctcgcaa
Mod(mdg4)	G2038	cagaagcaaagctccaccagcaat
	G2039	agtcaggttcagatttcgtgggca
eEF1d	G2040	aacgacgacgatgacgatgtggat
	G2041	ctttcttggccttcttggcagcat
